# Endocannabinoids and related lipids linked to social exclusion in individuals with chronic non-medical prescription opioid use

**DOI:** 10.1038/s41386-024-01881-8

**Published:** 2024-05-21

**Authors:** Sara L. Kroll, Philip Meier, Leah M. Mayo, Jürg Gertsch, Boris B. Quednow

**Affiliations:** 1 Social and Affective Neuropsychopharmacology, Adult Psychiatry and Psychotherapy, University Hospital of Psychiatry Zurich, University of Zurich, Zurich, Switzerland; 2 Experimental and Clinical Pharmacopsychology, Adult Psychiatry and Psychotherapy, University Hospital of Psychiatry Zurich, University of Zurich, Zurich, Switzerland; 3 Neuroscience Center Zurich, University of Zurich and Swiss Federal Institute of Technology, Zurich, Switzerland; 4 Institute of Biochemistry and Molecular Medicine, University of Bern, Bern, Switzerland; 5 Mathison Centre for Mental Health Research and Education, Hotchkiss Brain Institute, and Department of Psychiatry, University of Calgary, Calgary, AB Canada

**Keywords:** Translational research, Addiction, Stress and resilience

## Abstract

Opioid-related overdose deaths are still on the rise in North America, emphasizing the need to better understand the underlying neurobiological mechanisms regarding the development of opioid use disorder (OUD). Recent evidence from preclinical and clinical studies indicate that the endocannabinoid system (ECS) may play a crucial role in stress and reward, both involved in the development and maintenance of substance use disorders. Animal models demonstrate a specific crosstalk between the ECS and the endogenous opioid system. However, translational studies in humans are scarce. Here, we investigated basal plasma levels of the endocannabinoids anandamide (AEA) and 2-arachidonoyglycerol (2-AG), and eight endocannabinoid-related lipids, including oleoylethanolamide (OEA) and palmitoylethanolamide (PEA), as well as whole blood fatty acid amide hydrolase (FAAH) activity in chronic non-medical prescription opioid users (NMPOU; *n* = 21) compared to opioid-naïve healthy controls (*n* = 29) considering age, sex, and cannabis use as potential confounders. Additionally, the association of endocannabinoids and related lipids with the participants’ response to experimentally induced social exclusion was examined. We found significantly elevated basal AEA, OEA, and PEA levels in NMPOU compared to controls, but no differences in FAAH activity, 2-AG, or other endocannabinoid-related lipids. Within NMPOU, higher AEA levels were associated with lower perception of social exclusion. Robust positive correlations within *N*-acylethanolamines (i.e., AEA, OEA, and PEA) indicate strong metabolic associations. Together with our recent findings of elevated basal 2-AG levels in dependent cocaine users, present results indicate substance-specific alterations of the ECS that may have implications in the search for novel therapeutic interventions for these populations.

## Introduction

The opioid epidemic in North America has been exacerbated during the COVID-19 pandemic, reporting an increase of fatal opioid overdose rates up to 72% in Canada and 35% in the US from 2019 to 2020 [1, 2]. Despite the vast research in opioid addiction over the past decades, little is known about the neurobiological mechanism underpinning the development and maintenance of opioid use disorder (OUD). Therefore, opioid abstinence remains one of the major challenges in the treatment of OUD. The gold standard is still opioid substitution therapy (OST), targeting the opioid system by using opioid agonists and maintaining opioid dependence [3, 4]. However, the relapse rates following opioid detoxification and discontinuation of OST are higher than for any other substance, with rates up to 90% [5–7], highlighting a lack of effective treatments apart from lifetime opioid substitution and the need for novel treatments for OUD [4, 8].

Psychosocial stress is a well-known risk factor for the development and maintenance of substance use disorder (SUD), for example, by increasing substance craving [9–11]. Furthermore, substance use itself can lead to alterations in physiological and subjective stress responses, strengthening the vicious circle of SUD [12]. Endogenous or exogenous activation of the mu-opioid receptor (MOR) system by opioids has been repeatedly shown to induce stress-relieving effects in animal models, especially within social contexts, whereas MOR antagonists such as naltrexone showed opposite effects [13, 14]. Human studies consistently reported stress-dampening effects of acute opioid administration on physiological stress responses as measured by activity of the hypothalamic-pituitary-adrenal (HPA) axis [15–17]. However, acute opioid effects on subjective stress response in healthy human individuals are rather inconsistent, as either a reduction of subjective feelings of stress [16, 18, 19] or increased subjective stress responses [20] have been reported. Inconsistent findings were also reported for the few studies investigating subjective and physiological stress response in individuals with chronic opioid use, showing increased stress and anger, no effects, or dampened stress responses in chronic opioid users compared to healthy controls [21–24].

Preclinical studies suggest that the endocannabinoid system (ECS) plays a fundamental regulatory role in subjective and physiological stress responses. More precisely, stress-induced activation of the HPA axis has been closely linked to the ECS in animal models through the main endocannabinoid ligands anandamide (AEA) and 2-arachidonoyglycerol (2-AG), both activating the cannabinoid type-1 (CB1) receptors in brain regions involved in processing (affective) stress responses [25–28]. Moreover, elevated AEA levels, caused either by genetic or pharmacological inhibition of the degrading enzyme fatty acid amide hydrolase (FAAH) – which terminates AEA signaling –, resulted in stress-buffering effects in rodents, which has been recently also confirmed in humans [25, 29–31].

Interestingly, animal models indicate a crosstalk between the ECS and endogenous opioid system [32, 33]. Preclinical data support reciprocal interactions between both neurochemical systems on both anatomical and molecular levels [32, 34]. Accordingly, preclinical findings indicate that acute and chronic opioid administration can cause changes in the ECS, which may facilitate development and maintenance of OUD [32, 35]. However, only a few animal studies have tested the pharmacological effects of opioids on endocannabinoids and related lipids in preclinical addiction models, while human studies are missing so far. Despite consistent evidence of elevated AEA and decreased 2-AG levels after acute opioid administration in the nucleus accumbens (NAc) and other brain regions [36–38], but see also [39, 40], less is known about chronic opioid effects in animals. Moreover, the few existing preclinical findings of repeated opioid administration are difficult to interpret and less translatable to humans regarding alterations of endocannabinoid levels in OUD.

In sum, chronic effects of opioids on the ECS are less tested and alterations of the ECS in OUD remain poorly understood. Therefore, the aim of the present study was to investigate the ECS in chronic opioid users and whether this might be linked to subjective stress response of social rejection. Here, we analyzed plasma levels of the main endocannabinoid ligands AEA and 2-AG as well as other endocannabinoid-related lipids including *N*-acylethanolamines, as well as FAAH activity of previously collected data that includes individuals with NMPOU and controls [23]. Since preclinical findings show an opioid-endocannabinoid crosstalk [32] and acute opioid effects on endocannabinoids [35], we expected altered peripheral endocannabinoid levels in chronic opioid users compared to healthy controls. Finally, we hypothesized that endocannabinoid levels would be associated with the stress response to social rejection based on reported stress-dampening effects of endocannabinoids, especially AEA, in animals and humans [25, 30].

## Methods and materials

### Participants

Available plasma samples from a previously published study, where we analyzed stress reactivity of the HPA axis to social rejection [23], were used to analyze endocannabinoids and structurally or biochemically related lipids of individuals with NMPOU (*n* = 21) and opioid-naïve healthy controls (*n* = 29). Due to unexpected difficulties in drawing blood from two participants of the NMPOU group, we were able to include data of only 21 instead of initial 23 NMPOU group participants in the present analyses. Inclusion criteria for the NMPOU group were chronic non-medical use of prescription opioids over at least the last six months and no current or history of intravenous (i.v.) street heroin use or heroin dependence. Chronic NMPOU was determined by self-reports and objectively confirmed by toxicological hair and urine analysis (for technical details see ref. [41]).

General inclusion and exclusion criteria have been described in more detail previously [41]. Briefly, exclusion criteria were neurological disorders or head injuries, severe physical diseases, frequent cannabis use, severe psychiatric disorders such as post-traumatic stress disorder (except for alcohol and tobacco use disorders as well as former depressive episodes), chronic pain, and recent emotionally stressful and painful events. Participants were recruited through advertisements in internet forums, local newspapers, and through specialized addiction centers. All participants were instructed to abstain from psychotropic substances for 72 h and for 24 h from alcohol before onset of the test session. Furthermore, opioid users were asked to abstain from opioids on the testing day, or to take an adequate and minimized dose of opioids, if necessary, which solely removed withdrawal symptoms, to avoid measuring neither acute nor withdrawal effects. Recent use from opioids and other substances was controlled by urine analyses.

The study has been carried out in accordance with the Declaration of Helsinki and was approved by the Cantonal Ethics Committee of Zurich (KEK-Nr. 2015-0238). All participants provided written informed consent and were financially compensated for their participation.

### Procedure

All test sessions started around 11am with a screening for psychiatric disorders by using the Structured Clinical Interview for axis-I DSM-IV Disorders (SCID- I; [42]), adapted for DSM-5 regarding SUD. Self-report of substance use was examined by the standardized and structured Interview for Psychotropic Drug Consumption [43]. Eligible participants were then screened for severity of nicotine dependence using the Fagerström Test of Nicotine Dependence (FTND; [44]), for depressive symptoms assessed by the Beck Depression Inventory (BDI; [45]), and premorbid verbal IQ using a German vocabulary test – the Mehrfachwahl-Wortschatz-Intelligenztest (MWT-B; [46]). Within the NMPOU group, the following opioid use variables were assessed: Current opioid craving assessed by a Numeric Rating Scale (NRS) from 1 (no craving) to 10 (highest craving) and opioid withdrawal symptoms using the Objective Opioid Withdrawal Scale (OOWS; [47]). After the assessments, an i.v. catheter was placed in the forearm vein of the non-dominant hand. The whole blood sample was collected when the i.v. catheter was placed and the plasma sample was collected approximately one hour after the placement around 1 pm, directly before onset of the social stressor. Following the social exclusion task additional neuropsychological tests were conducted, which have been reported elsewhere [41, 48].

### Social stress task

The Cyberball task has been shown to robustly induce negative affect and feelings of social exclusion [49–51]. It is a virtual ball-tossing task with two other players. To increase credibility of the game, the two players were personally introduced before the Cyberball onset and photos of all the players including the participant were shown in the task. The total duration of the task was three minutes (60 throws). First, participants were included in the game for about one minute receiving the ball six times (10%). This was followed by the exclusion condition, where the participant did not receive the ball anymore for the next two minutes, as controlled by the computer. Negative affect to social rejection was measured by the Positive and Negative Affect Schedule (PANAS; [52]) before and after the Cyberball and change scores were used for analyses. After the Cyberball task, participants were asked to estimate how often they received the ball (in percentage) and how excluded as well as included they felt during the whole task on a nine-point Likert scale.

### Quantification of endocannabinoids and associated lipids

For the quantification of endocannabinoids and associated lipids in plasma samples, an earlier validated and published method was applied using a liquid-liquid extraction followed by liquid chromatography-electrospray ionization-tandem mass spectrometry (LC-ESI-MS/MS) analysis [53]. The two endocannabinoids 2-AG and AEA, as well as the *N*-acylethanolamines linoleoyl ethanolamide (LEA), oleoylethanolamide (OEA), palmitoylethanolamide (PEA), and stearoyl ethanolamide (SEA), and endocannabinoid-related lipids 1,2-diarachidonoyl-sn-glycero-3-phosphoethanolamine (20:4PE), 1-Stearoyl-2-arachidonoyl-sn-glycerol (SAG), 2-oleoylglycerol (2-OG) and arachidonic acid (AA) were quantified. For a detailed description see Supplementary Materials Method S1 and Supplementary Table S1.

### Analysis of AEA hydrolysis in whole blood

The analysis of general and FAAH-mediated AEA hydrolysis in blood was quantified by measuring the hydrolysis of the radioactive ethanolamine-1-3H AEA (^3^H-AEA) in lysed whole blood samples of the NMPOU and control group with or without addition of the FAAH inhibitor URB597. For detailed description see Supplementary Materials Method S2. The FAAH-mediated hydrolysis (FAAH activity) for each participant was calculated as percentage of the overall AEA hydrolysis as follows: 100–(100/%AEA hydrolysis of DMSO samples*%AEA hydrolysis of URB597 samples).

### Statistical analyses

Statistical analyses were performed with IBM SPSS Statistics 28.0.1.1 or GraphPad Prism 10.1.0 (GraphPad Software, USA). Pearson’s *χ*^2^-tests were carried out to analyze frequency data. Quantitative between-group data were analyzed either by independent *t*-tests or Mann–Whitney-U-tests for non-normally distributed data. For the primary outcome variables of basal plasma concentrations of endocannabinoids and related lipids, analyses of covariance (ANCOVAs) were performed with *GROUP* (controls, NMPOU) as the fixed factor to control for the reported confounding variables sex [54] and age [55]. To test whether differences in plasma concentrations of endocannabinoids and related lipids were associated with opioid use intensity (see Table 1), we used Spearman’s rank correlations within the NMPOU group due to its robustness to outliers, skewed distributions, and small sample sizes.

**Table 1:** Demographic data and drug use (means and standard deviations).

	Controls	NMPOU	Value	*df*	*p*
	(*n* = 29)	(*n* = 21)			
Female/male	10/19	5/16	χ^2^ = 0.66	1	0.416
Age	26.6 (8.1)	28.7 (10.4)	t = −0.83	48	0.411
Years of education	11.5 (1.5)	11.1 (2.0)	t = 0.67	35.6	0.507
Ethnicity					
White/European	29	17			
Black/African-European^a^	0	2			
Asian	0	0			
Hispanic/Latino^a^	0	2			
Verbal IQ	105.2 (11.3)	106.4 (11.2)	t = −0.37	50	0.717
BDI sum score	3.0 (3.3)	9.8 (7.9)	t = −4.16	48	**<0.001**
BMI	22.2 (2.6)	24.1 (3.5)	t = −1.95	48	0.058
Smoking y/n	18/11	15/6	χ^2^ = 0.48	1	0.490
Cigarettes/week^b^	45.4 (35.7)	87.8 (57.7)	t = −2.50	22.5	**0.021**
Alcohol y/n	27/2	19/2	χ^2^ = 0.11	1	0.735
Alcohol gram/week^b^	74.8 (64.9)	61.0 (64.4)	t = 0.71	44	0.479
Opioid use					
Times per week	–	3.9 (3.1)			
ME mg/week	–	586.2 (1001)			
Years of use^c^	–	2.9 (0.5–28.0)			
Last opioid used in hours^c^	–	24.0 (1–729.6)			
OUD in percentage^d^		76			
Craving (NRS)	–	3.3 (2.6)			
Opioid withdrawal (OOWS)	–	1.0 (2.1)			
Positive urine tests y/n	0/29	10/11			
ME hair concentration pg/mg	1 (4)	5084 (7314)			
Cannabis use					
Cannabis use y/n	9/20	13/8	χ^2^ = 4.71	1	**0.030**
g/week^b^	0.21 (0.5)	0.62 (0.7)	U = 25.0		**0.025**
Years of use^b^	4.50 (3.1)	1.87 (1.6)	U = 24.0		**0.020**
Positive urine tests (y/n)	0/29	5/16			

Significant *p*-values *(p* < 0.05) are shown in bold. *T*-test and Mann–Whitney U test for quantitative data, Chi^2^ for frequency data.

*BDI* Beck’s Depression Inventory, *BMI* Body Mass Index, *ME* morphine equivalent, *NRS* numeric rating scale (1–10), *OOWS* objective opioid withdrawal scale (0–12), *OUD* Opioid Use Disorder.

^a^At least one parent with this ethnically background.

^b^Only within users.

^c^Median (range) are reported.

^d^Fulfilling DSM 5 criteria of OUD (moderate to severe).

For our secondary outcome, we used Linear Mixed Models (LMM) to determine whether feelings of social exclusion as well as changes in negative affect in response to the social stress task can be predicted by *basal endocannabinoids and related lipids* and its interaction with *GROUP*, with the additional fix factors *SEX* and *AGE* and their interactions to control for confounding effects. Post hoc Spearman’s rank correlations within each group were performed for significant interaction effects, to formally test whether the results of the LMM were unique to the NMPOU group.

To determine associations of the quantified lipids in plasma, as well as between FAAH-mediated AEA hydrolysis in whole blood and lipid plasma levels, additional Spearman’s rank correlations were performed.

Cohen’s *d* effect size was calculated by the means and pooled standard deviations of the groups [56].

The statistical comparisons were carried out with a significance level of *p* < 0.05 (two-tailed).

## Results

Participants’ demographic and substance use data including opioid use variables are shown in Table 1.

### *N*-acylethanolamines and endocannabinoid-related lipids

**Fig. 1:**
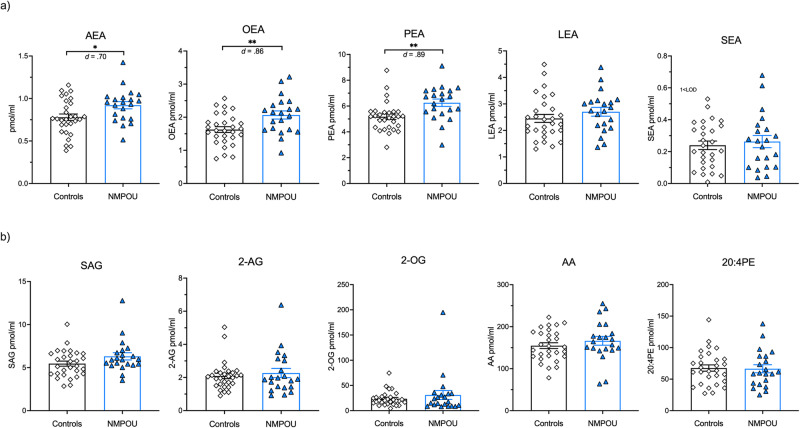
Elevated *N*-acylethanolamines in chronic opioid users. Individuals with chronic non-medical prescription opioid use (NMPOU; blue; triangles) showed significantly higher basal plasma levels of (**a**) anandamide (AEA), palmitoylethanolamide (PEA), and oleoylethanolamide (OEA) compared to drug-naïve healthy controls (white; diamonds), but no group difference was found for (**b**) 1-Stearoyl-2-arachidonoyl-sn-glycerol (SAG), linoleoyl ethanolamide (LEA), stearoyl ethanolamide (SEA), 2-arachidonylglycerol (2-AG), 2-oleoylglycerol (2-OG), arachidonic acid (AA). Bars represent means including individual data points, and error bars reflect standard error of the mean (SEM); corrected for age and sex. Significant *p*-values marked with *p*-values < 0.05*, *p*-values < 0.01**.

ANCOVAs including sex and age as covariates and the dependent variables endocannabinoid and related lipids yielded significant *GROUP* effects for the *N*-acylethanolamines AEA, OEA, and PEA, but not LEA and SEA (*p*-values > 0.227). More precisely, the NMPOU group showed elevated levels of AEA (F(1,46) = 6.17, *p* = 0.017, *d* = 0.70), OEA (F(1,46) = 9.66, *p* = 0.003, *d* = 0.86), and PEA (F(1,46) = 9.42, *p* = 0.004, *d* = 0.89) compared to healthy controls (see Fig. 1a). No differences were found for 2-AG (F(1,46) = 0.18, *p* = 0.894, *d* = 0.17) and endocannabinoid-related lipids (*p*-values > 0.166; see Fig. 1b). These results were robust to a correction for multiple comparisons using a False Discovery Rate (FDR) approach (see Supplementary Table S2) [57]. To consider potential confounding effects of cannabis use and depressive symptoms, we added the variables *cannabis use within the last six months (yes/no)* and the *BDI sum score* as covariates into the analyses, which did not change the main results (see Supplementary Table S3a, and S3b, respectively).

No significant correlations were found between *N*-acylethanolamines and opioid use intensity (*p*-values > 0.121; see Supplementary Fig. S1). *N*-acylethanolamines did also not differ between individuals with positive and negative opioid urine sample within the NMPOU group (*p*-values > 0.187). Moreover, no correlations between *N*-acylethanolamines and alcohol, tobacco, and cannabis use variables were found except for *cigarettes used per week* over all tobacco users (see Supplementary Table S4). However, adding *cigarettes used per week* as a covariate into the ANCOVAs did not change our main findings (see Supplementary Table S3c).

### Fatty acid amide hydrolase activity

To formally test whether our results of elevated plasma *N*-acylethanolamine concentrations are related to circulating cells, which can release and hydrolyze *N*-acylethanolamines [58], or may reflect changes of tissue ECS metabolism, we determined the hydrolysis of ^3^H-AEA in lysed whole blood samples. As shown in Fig. 3b, independent *t*-tests yielded no group differences in the general and FAAH mediated hydrolysis of ^3^H-AEA (*p*-values > 0.126). Results remained non-significant even after controlling for sex and age (*p*-values > 0.177). However, trends for a negative association between the FAAH activity in whole blood and the plasma *N-*acylethanolamines AEA and OEA, as well as AA (*p*-values < 0.097) of NMPOU but not controls were observed. In addition, a bimodal distribution of the whole blood FAAH activity was observed in the control but not NMPOU group (Fig. 3b). To further investigate, we performed a median split over all individuals (median 16.7 FAAH activity) to distinguish between high/low FAAH activity within each group. Within low FAAH activity, concentrations of the AEA, OEA, and PEA were significantly higher in the NMPOU compared to the control group (Fig. 3c), whereas no differences were found for other endocannabinoid-related lipids (Supplementary Fig. S2). Within high FAAH activity subgroups, no differences of *N*-acylethanolamines between NMPOU and controls were found. Further, AA was significantly higher in plasma of NMPOU with low whole blood FAAH activity compared to plasma of NMPOU with high whole blood FAAH activity. This was not observed in controls (Fig. 3c).

### Social exclusion and *N*-acylethanolamines

The LMM results for “estimated % balls received” yielded only one significant effect, which is the interaction effect of AEA*GROUP (F(1,50) = 9.96, *p* = 0.003, *η*_*p*_^*2*^ = 0.17). For the dependent variable “feeling included”, again only the interaction effect *AEA* *×* *GROUP* (F(1,50) = 7.06, *p* = 0.011, *η*_*p*_^*2*^ = 0.12) was significant. The LMM results for “feeling excluded” showed a trend level for AEA*GROUP interaction (F(1,50) = 2.90, *p* = 0.095, *η*_*p*_^*2*^ = 0.05). Similar effects were found for OEA*GROUP interaction on “estimated % balls received” (F(1,50) = 11.87, *p* = 0.001, *η*_*p*_^*2*^ = 0.19) and “feeling included” (F(1,50) = 4.13, *p* = 0.047, *η*_*p*_^*2*^ = 0.08). LMMs including the fixed factor PEA were not significant (*p*-values > 0.230). No significant AEA/OEA/PEA*GROUP interactions were found for the change score in positive (PA) and negative affect (NA) of the PANAS as dependent variables (*p*-values > 0.207). As previously reported, no main effect of *GROUP* was found for feelings of exclusion after the social stress task as well as changes in affect [23]. All LMMs for AEA, OEA, and PEA are reported in the Supplementary Table S5a, S5b, and S5c, respectively. Furthermore, we added *cannabis use* and *depressive symptoms* as potential confounding variables into the models reported in the Supplementary Tables S6a–c and S7a–c, respectively, which did not change the results.

**Fig. 2:**
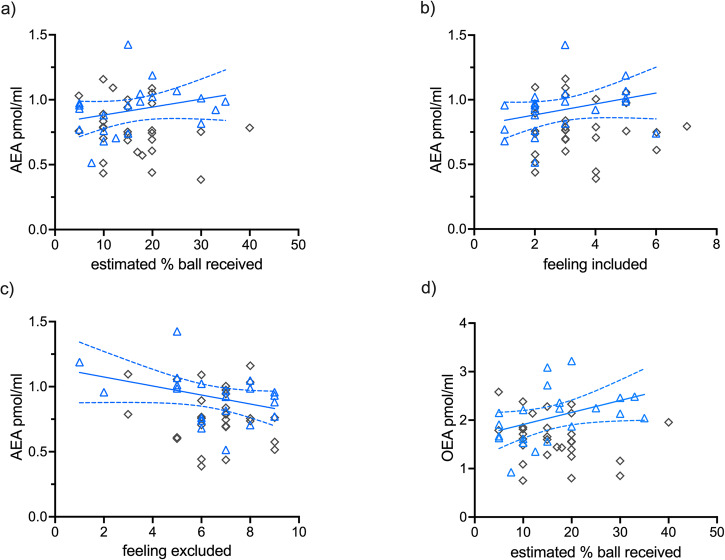
Spearman’s correlation for *N*-acylethanolamines and social stress variables. Significant spearman’s correlations were only found within NMPOU (blue triangles) for: **a** AEA plasma levels and *estimated % of balls received* and **b** *feeling included*; **c** trend level effects for *feeling excluded* (r(21) = −0.41, *p* = 0.068); **d** significant correlation between OEA and *estimated % of balls received*. No significant correlations were found within controls (gray diamonds) *p*-values > 0.299.

Post hoc correlation analyses for significant AEA*GROUP interactions showed that these effects were specifically unique to the NMPOU group with moderate correlations between AEA and “estimated % balls received” (r_s_(21) = 0.5 *p* = 0.041), “feeling included” (r_s_(21) = 0.5, *p* = 0.035), and “feeling excluded” (r_s_(21) = −0.4, *p* = 0.068), whereas no significant correlations were found for the control group (*p*-values > 0.299) as shown in Fig. 2a–c. Fisher’s r-to-z transformation comparing correlation coefficients showed significant group differences for “estimated % balls received” (Z = 2.24, *p* = 0.025). A moderate to strong positive correlation was found for OEA and “estimated % balls received” within the NMPOU group (see Fig. 2d). No further significant correlations were found for OEA and PEA.

### Correlations of plasma lipid levels and fatty acid amide hydrolase activity

**Fig. 3:**
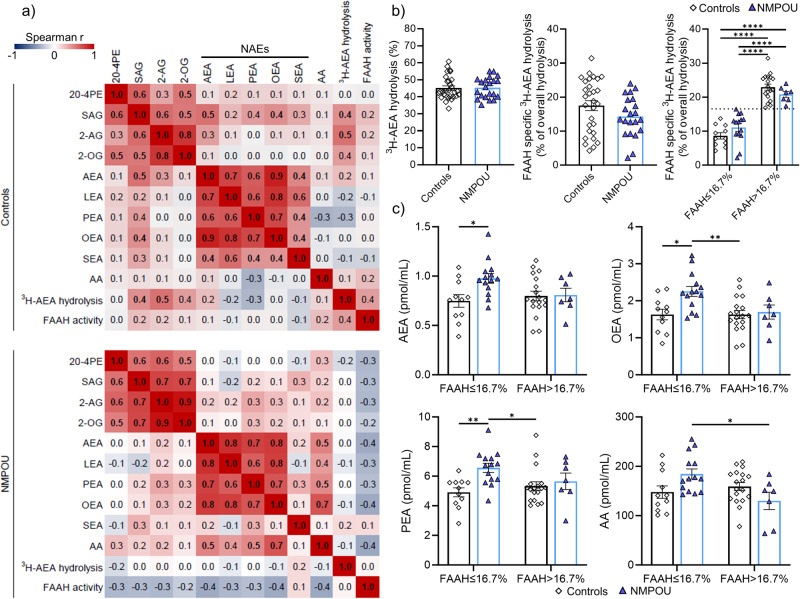
Correlation patterns of plasma lipids and analysis of general and FAAH specific hydrolysis of ^3^H-AEA in whole blood samples of controls and NMPOU. **a** Spearman correlation heatmaps of quantified lipid levels in plasma and general, as well as FAAH specific hydrolysis (FAAH activity, see **b**) of ^3^H-AEA in lysed whole blood samples. For each pairwise correlation the Spearman r is shown in the heatmaps. The color-coding legend of the Spearman r values is presented and is reaching from −1 (strong negative correlation, blue) over 0 (no correlation, white) to 1 (strong positive correlation, red). Significant correlations (*p* < 0.05) are highlighted with bold Spearman r values. **b** General and FAAH mediated hydrolysis (FAAH activity) of ^3^H-AEA in whole blood samples, as well as whole blood FAAH activity of participants sorted into groups with high and low FAAH activity based on median split of all participants (median FAAH activity 16.7%). **c** Selected graphs of quantified plasma *N*-acylethanolamines and AA levels sorted by FAAH activity quantified in whole blood (see **b**, FAAH specific ^3^H-AEA hydrolysis). **b**, **c** Data are presented as scatter dot plot with bar showing mean ± SEM. Each dot represents the quantified value of one participant. For statistical analysis a two-tailed, unpaired *t*-test was performed with a *p*-value < 0.05 considered to indicate a significant difference between the two compared groups. Significant *p*-values marked with *p*-values < 0.05*, *p*-values < 0.01**, *p*-values < 0.001***, *p*-values < 0.001***. 20:4PE 1,2-diarachidonoyl-sn-glycero-3-phosphoethanolamine, 2-AG 2-arachidonoylglycerol, 2-OG 2-oleoylglycerol, AA arachidonic acid, AEA arachidonoyl ethanolamide, FAAH fatty-acid amide hydrolase, LEA linoleoyl ethanolamide, NAEs *N*-acylethanolamines, OEA oleoylethanolamide, PEA palmitoylethanolamide, SAG 1-Stearoyl-2-arachidonoyl-sn-glycerol, SEA stearoyl ethanolamide.

Spearman’s rank correlation coefficients within the NMPOU and control group are shown in Fig. 3a. Correlation analysis showed moderate to strong positive correlations of the endocannabinoid 2-AG with its precursor lipid SAG, as well as with the structurally related lipid 2-OG in the plasma samples of both groups. In addition, significant positive associations of all quantified *N*-acylethanolamines were observed in controls. While the *N*-acylethanolamines AEA, LEA, OEA and PEA were positively correlated in plasma of NMPOU, SEA was not significantly associated with the other *N*-acylethanolamines. On the other hand, a significant positive association of the *N*-acylethanolamines AEA, PEA and OEA was found with AA in plasma of NMPOU participants, but not in controls.

## Discussion

Our results provide evidence of altered blood ECS associated with chronic NMPOU in humans. Peripheral levels of *N*-acylethanolamines (i.e., AEA, PEA, and OEA) were elevated in the NMPOU group compared to opioid-naïve healthy controls, whereas no group differences for 2-AG and other endocannabinoid-related lipids were found. Additional whole blood analysis showed neither group differences of FAAH activity nor an association between FAAH and peripheral *N*-acylethanolamines levels in our samples, indicating that these plasma *N*-acylethanolamine concentrations cannot be explained by blood cell degradation. This might suggest that *N*-acylethanolamines are differentially generated in the tissues or that FAAH activity in organs is affected. Elevated basal AEA levels were associated with less feelings of experimentally induced social rejection in the NMPOU group but not in healthy controls. This finding is interesting as it supports animal models on the stress dampening effects and synergy between the ECS and endogenous opioid system [25, 32, 34, 59].

Despite the relatively small sample size of the NMPOU group, we found robust effects with moderate to strong effect sizes for elevated AEA, OEA, and PEA plasma levels in individuals chronically stimulating their MOR system compared to opioid-naïve controls. MOR and CB1 receptors are both Gi/o-coupled receptors, which are similarly distributed throughout the body and often co-localized in striatal GABAergic neurons in the brain [32, 34]. Their cellular co-expression may lead to MOR and CB1 heterodimer formation and activation of these receptor complexes subsequently result in synergistic inhibition of neurotransmitter release underpinning the cannabinoid-opioid crosstalk [32, 34]. Although OUD has been linked to alterations of the ECS in animals [60], only few preclinical studies, and no human studies, have tested the pharmacological effects of opioids on endocannabinoid levels so far [35]. Whereas preclinical data consistently showed increased AEA levels after acute morphine and heroin administration regardless of prior opioid exposure [36–38], but see [39], no differences in endocannabinoid levels after repeated opioid administration were found [36, 38–40]. Of note, most of the studies used non-contingent drug administration methods lacking animal-driven behavior [36, 37, 39, 40], which may reflect the initial phase of drug intake rather than the transition to compulsive substance use disorder [61].

Increased substance-taking and seeking behavior are key features of SUD in humans, commonly addressed by self-administration (SA) paradigms in animal models of addiction [62, 63]. Yet only one study has investigated the effects of opioid addiction on endocannabinoids using 2-hour-sessions of heroin SA [38]. Although no differences were found in AEA and 2-AG in rats with previous heroin SA history compared to drug-naïve animals (but also no differences for ethanol and cocaine SA), AEA significantly increased acutely during heroin SA, which was further positively correlated with the amount of heroin administered. Of note, the short-access paradigm used in this study may only address recreational substance use but not uncontrolled and compulsive substance-seeking behavior observed in long-access SA models, which are key features of SUD in humans [64]. Although some individuals of our NMPOU sample also showed recreational opioid use, 76% of the sample fulfilled the DSM-5 criteria for moderate to severe OUD. Importantly, our NMPOU sample showed volitional and uncontrolled opioid use in contrast to reported animal studies. Therefore, existing preclinical findings of chronic opioid effects on the ECS may not directly translate to humans, highlighting the need for further research to elucidate the role of the ECS in OUD and to confirm our findings.

Based on the reported acute effects of opioids on AEA, our findings in individuals with NMPOU might suggest that volitional chronic opioid use is associated with an upregulation of *N*-acylethanolamines including AEA signaling in humans. However, we did not find associations between *N*-acylethanolamines and opioid use intensity indicating acute opioid effects. Moreover, one inclusion criterion for the NMPOU group was regular opioid use for at least the last six months. Therefore, we speculate that our results of elevated *N*-acylethanolamine levels might be rather related to general chronic opioid use effects than to acute effects. Importantly, the present study was a cross-sectional study, therefore we cannot rule out the possibility that individuals with NMPOU might have preexisting elevated levels of *N*-acylethanolamines, which may lead to increased vulnerability to OUD. Additional preclinical studies are needed to back-translate our findings, using more consistent approaches as well as translational addiction models of OUD in animals to support our assumption and to provide reliable results regarding a potential up-regulation of AEA signaling in OUD.

Of note, we were only able to measure plasma levels of endocannabinoids and related lipids, which does not allow direct conclusions about brain-specific concentrations. However, given that lipids such as endocannabinoids can readily cross the blood-brain-barrier, peripheral plasma endocannabinoid levels are suggested as a reliable proxy of central endocannabinoid dynamics [58, 65]. Accordingly, recent translational findings indicate that peripheral endocannabinoid concentrations, genetically manipulated by the FAAH polymorphism, reflect brain levels in animals and humans [66], but see also [67]. Moreover, negative correlations between plasma levels of *N*-acylethanolamines and FAAH levels in the human brain using the FAAH tracer [^11^C]CURB in patients with alcohol use disorder [68] has been shown supporting the suggestion of peripheral endocannabinoids as a proxy for central endocannabinoid levels.

In line with the cannabinoid-opioid crosstalk, our findings may indicate potential stress-buffering effects of elevated AEA to social exclusion specifically related to chronic stimulation of the MOR system. This is further in line with consistent findings in preclinical and recent human studies showing stress-buffering effects of elevated AEA levels [25, 29–31]. Interestingly, in our previous study with the same NMPOU and control sample, we were not able to find group effects in self-reports of stress and feelings of exclusion [23]. This was in contrast to our initial hypothesis expecting that the NMPOU group would be less affected by social exclusion and show attenuated stress response, based on earlier animal studies consistently reporting stress buffering effects of opioids [13, 14, 69, 70]. However, results from human studies testing acute and chronic opioid effects on subjective stress response are quite heterogenous and inconsistent [16, 19–24]. Therefore, our findings of an association between elevated AEA and attenuated feelings of exclusion may suggest that not only activation of the MOR itself, but the interaction with the ECS might entail stress relieving effects of opioids, which may further explain the reported inconsistencies in human opioid studies so far. However, we cannot provide any mechanistic data or causality for this hypothesis with the present study. Moreover, previous preclinical findings suggest that oxytocin transmission increases AEA signaling in the NAc and regulate social interaction in mice [71]. Future studies exploring the interaction between the endogenous opioid, cannabinoid, and oxytocinergic system will be of high interest for improving our understanding of psychopathologies showing impaired social reward such as SUD and autism spectrum disorder.

Although correlations with such small sample sizes have to be interpreted carefully, correlation coefficients for the “estimated % of balls received”, as a proxy of feeling less excluded, were significantly different between groups. Given that stress and poor social support are key risk factors for SUD relapse [9, 72], and current OST medications act all on the opioid system, our findings might suggest that pharmacologically targeting the ECS, especially elevating AEA levels by FAAH inhibitors, may be a novel treatment option of OUD that could result in reduced stress-induced craving, promoting abstinence and preventing OUD relapse without maintaining opioid dependence. FAAH inhibitors have been extensively tested in preclinical models of addiction [60, 73, 74] showing attenuating effects on opioid withdrawal symptoms [75, 76], and in mice a genetic loss or inhibition of FAAH in the CNS was further reported to prevent and reverse morphine tolerance [77]. However, only a single clinical trial in SUD has been published so far, reporting beneficial effects of the FAAH inhibitor PF-04457845 in individuals with cannabis use disorder by reducing cannabis withdrawal symptoms and social anxiety [78]. Interestingly, FAAH knock-out mice showed increased reward-seeking behavior in the operant sensation-seeking paradigm [79] indicating an increase in dopamine-modulated reward behavior including natural rewards. Accordingly, FAAH knock-out mice showed significantly higher levels of social conditioned place preference (CPP) compared to wild-type littermates, whereas no differences in the CPP were found for high-fat food and cocaine [71]. Therefore, inhibition of FAAH might not only reduce withdrawal symptoms but also increase behavior towards natural and social rewards including social interaction, which is crucial to prevent relapse [80, 81]. Furthermore, activation of CB1R has been shown to reinstate cocaine-seeking [82], whereas CB1R antagonism was reported to block stress-potentiated reinstatement of cocaine-seeking behavior [83] indicating substance-specific involvement of the ECS in stimulant and opioid use disorder.

The biosynthesis of *N*-acylethanolamines is facilitated in the same enzymatic pathways and beside FAAH only a few other enzymes have been described to hydrolyze *N*-acylethanolamines in a tissue- or cell-specific manner [84]. Accordingly, positive correlations between the analyzed plasma *N*-acylethanolamines were observed, indicating a high metabolic association of *N*-acylethanolamines. In brain tissue, FAAH is regarded as the primary *N*-acylethanolamine hydrolyzing enzyme [84–87]. Genetic loss and pharmacological inhibition of FAAH led to increased AEA, PEA, and OEA levels in rodent brains [84, 87–90]. The FAAH inhibitor PF-04457845 has further been shown to reduce FAAH activity also in blood leukocytes and to increase AEA levels in plasma [89]. We did not find significant differences in general and FAAH specific ^3^H-AEA hydrolysis in whole blood between groups. Furthermore, the finding that circulating *N*-acylethanolamine levels of the controls were not correlating with FAAH activity in whole blood might indicate that the concentrations of *N*-acylethanolamines in the circulation are probably stronger influenced by release or uptake of *N*-acylethanolamines from various tissues [for review see 58] or by regulation of synthesis and release of *N*-acylethanolamines from blood cells rather than hydrolysis via FAAH in circulating blood cells. However, in NMPOU participants, we found trends for negative correlations of plasma *N*-acylethanolamine levels with whole blood FAAH activity and the plasma levels of AEA, PEA, and OEA were specifically increased in NMPOU with lower whole blood FAAH activity. The low numbers of study participants especially in the subgroups require a cautious data interpretation. Nevertheless, our observations suggest that opioids might cause a higher release or reduced uptake of circulating *N*-acylethanolamines by tissues or circulating cells, which leads to an accumulation of AEA, PEA, and OEA especially in opioid users with lower peripheral FAAH activity. Of note, the FAAH variant P129T, were a missense mutation (385C → A) leading to a reduced FAAH expression and activity, was earlier associated with higher vulnerability to different SUDs [91]. However, a previous study in heroin users and controls with different ethnicities did not find an association between 385C → A and heroin use disorder, whereas the gene polymorphism for the cannabinoid receptor 1 (CNR1) was found to be associated with heroin addiction [92]. Nevertheless, future studies should investigate if the increase of *N*-acylethanolamines in plasma of NMPOU observed in our study is especially associated with opioid users carrying the FAAH P129T variant.

While we found a significant increase of the endocannabinoid AEA and the associated *N*-acylethanolamines OEA and PEA, we could not find any relevant differences in 2-AG plasma levels between NMPOU and control participants. In animal studies with acute and chronic opioid administration, decreased concentrations of 2-AG in different brain regions have been reported [36–38, 40], but also no alterations of AEA and 2-AG [39]. However, our results indicate that such findings from animal studies seem not to translate to the circulating concentration of 2-AG in human NMPOU. This was in line with the comparable plasma concentrations of SAG, the precursor lipid of 2-AG, between both groups as the endocannabinoids 2-AG and AEA do not share the main metabolic pathways involved in their synthesis and degradation [93]. Therefore, our findings may show a specific impact of chronic opioid use on the metabolism of AEA and associated *N*-acylethanolamines but not 2-AG in the periphery.

In sum, our results indicate that *N*-acylethanolamines, especially AEA, may play a modulatory role in OUD in humans. Together with our recent findings in cocaine dependent individuals showing elevated 2-AG levels but no differences in *N*-acylethanolamines [94], this may indicate substance-specific alterations and involvements of the ECS in SUD suggesting different psychopharmacological targets within the ECS as potential treatments for stimulant use disorder and OUD. The association of elevated AEA levels and reduced social stress, which was only evident in individuals with NMPOU, suggests that *N*-acylethanolamines, including AEA, could be involved in OUD. Future studies using pharmacological manipulations to increase AEA levels through FAAH inhibition, or selective endocannabinoid reuptake inhibitors, in individuals with OUD are needed and will provide important information about its potential use as a novel treatment for OUD.

### Supplementary information

Supplementary Materials

## Data Availability

The data that support the findings of this study are not openly available due to reasons of sensitivity and are available from the corresponding author upon reasonable request.
