# Supplementary material for: Endocannabinoids and related lipids linked to social exclusion in individuals with chronic non-medical prescription opioid use
Source: Neuropsychopharmacology. 2024 May 21;49(10):1630–9. doi: 10.1038/s41386-024-01881-8 (PMC11319498; doi:10.1038/s41386-024-01881-8)
Supplement: Supplementary file 1 — Supplementary Materials [file 41386_2024_1881_MOESM1_ESM.docx]

**Supplementary materials**

**Endocannabinoids and related lipids linked to social exclusion in individuals with chronic non-medical prescription opioid use**

Sara L. Kroll, Philip Meier, Leah M. Mayo, Jürg Gertsch, Boris B. Quednow

**Method S1: Quantification of endocannabinoids and associated lipids in plasma samples of NMPOU and control participants**

For the quantification of endocannabinoids and associated lipids in plasma samples, an earlier published method was applied using a liquid-liquid extraction followed by liquid chromatography-electrospray ionization-tandem mass spectrometry (LC-ESI-MS/MS) analysis [1]. In brief, of each participant 200 µL of plasma were added to 0.75 mL of ethyl acetate:hexane (9:1, HPLC grade, VWR Chemicals, Poland) with 0.1 % formic acid (Sigma-Aldrich Chemie GmbH, Germany) already containing 1 µL of an internal standard mixture prepared in absolute ethanol (VWR International, France). After addition of 50 µL 0.1 M formic acid, the samples were strongly vortexed and sonicated for five minutes in a pre-cooled water bath followed by a centrifugation for 10 minutes with 1620 g at 4°C. The samples were located on dry ice for 10 minutes to freeze the lower aqueous phase and the upper organic phase was collected and evaporated in plastic tubes for 45 min using an Eppendorf Concentrator plus (Eppendorf AG, Germany). The dried pellets were reconstituted in 35 µL of Acetonitrile:H_2_O (8:2, HPLC grade, Fisher Scientific, UK) and 10 µL of the resuspended samples were injected into the LC-ESI-MS/MS system (linear ion trap quadrupole LC-MS/MS mass spectrometer, QTRAP 5500, AB Sciex Instruments, Massachusetts, USA). The LC-ESI-MS/MS conditions were used as described before [1] and the analytes 1,2-diarachidonoyl-sn-glycero-3-phosphoethanolamine (20:4PE), 1-Stearoyl-2-arachidonoyl-sn-glycerol (SAG), 2-arachidonoylglycerol (2-AG), 2-oleoylglycerol (2-OG), AEA, linoleoyl ethanolamide (LEA), oleoylethanolamide (OEA), palmitoylethanolamide (PEA), and stearoyl ethanolamide (SEA) were analysed using the turbo-ion spray interface operated in positive mode, while arachidonic acid (AA) was analysed with the turbo-ion spray interface operated in negative mode. The used MRM transitions and internal standards for each analyte are summarized in the supplementary table below. For the quantification a calibration curve was used with increasing amounts of analyte standards in a matrix of 1 % bovine serum albumin (fatty acid free, Sigma-Aldrich, Missouri, USA) in ddH_2_O. The processing and the measurements of the calibration samples was performed in the exact same way as the plasma samples. A linear calibration curve for each analyte with the quantified area ratio (analyte peak area/internal standard peak area) and the known analyte concentration was generated with the software Analyst 1.6.3 (AB Sciex, Massachusetts, USA) and the concentration of each analyte was calculated for the sample preparations. To determine the analyte concentration in plasma, the quantified amount of each analyte was normalized to the sample volume (200 µL) used for the extraction. 2-AG can isomerize to 1-AG with several different storage and extraction methods [2]. In the chromatography 1-AG is released at a slightly different retention time leading to a second peak in the chromatogram. To analyse the biological levels of 2-AG in the plasma samples the peaks of 2-AG and 1-AG were quantified together.

**Table S1:** Monitored MRM transitions and used internal standards of all quantified analytes.

| **Analyte (m/z)** | **Internal standard (m/z)** |
| --- | --- |
| 20:4PE (788.5 → 361.2) | 2-AG-*d5* (384.1 → 287.2) |
| SAG (667.4 → 327.1) | 2-AG-*d5* (384.1 → 287.2) |
| \| 2-AG (379.1 → 287.2) \| \| --- \| | 2-AG-*d5* (384.1 → 287.2) |
| 2-OG (357.3 → 265.3) | 2-AG-*d5* (384.1 → 287.2) |
| AEA (348.3 → 62.1) | AEA-*d4* (352.2 → 66.0) |
| LEA (324.2 → 66.9) | LEA-*d4* (328.1 → 66.0) |
| OEA (326.3 → 309.2) | OEA-*d4* (330.3 → 66.0) |
| PEA (300.2 → 282.2) | PEA-*d4* (305.0 → 62.0) |
| SEA (328.0 → 311.0) | AEA-*d4* (352.2 → 66.0) |
| AA (303.1 → 59.0) | AA-*d8* (311.1 → 59.0) |

**Method S2: Analysis of AEA hydrolysis in whole blood of NMPOU and controls**

Whole blood samples collected in EDTA tubes (BD Vacutainer) were thawed and kept cool on ice. The blood cells were lysed using an ultrasonic processor UP50H (Hielscher Ultrasonic GmbH, Germany) for 20 seconds with an amplitude of 100 % and an interval of 0.5 seconds. The whole blood lysates were afterwards aliquoted, snap frozen in liquid nitrogen and stored at -80 °C. The analysis of AEA hydrolysis in the whole blood lysate of each participant was performed in doublets for each condition. Therefore, 245 µL of the whole blood lysates were incubated for 15 minutes with 2.5 µL DMSO or URB597 (100 µM in DMSO, final concentration 1 µM, Cayman Chemical Company, USA) on a Thermomixer (Eppendorf AG, Germany) shaking with 400 rpm at 37 °C. Afterwards, 2.5 µL of 50 nM ethanolamine-1-3H AEA (American Radiolabeled Chemicals Inc., USA) and 9.95 µM AEA (Cayman Chemical Company, USA) in absolute ethanol was added to each sample and incubated for 30 minutes on a Thermomixer with 400 rpm at 37 °C. The AEA hydrolysis was stopped by addition of 500 µL of pre-cooled Chloroform/Methanol (1:1, Biosolve Chimie SARL, France; Dr. Grogg Chemie AG, Switzerland) and the samples were centrifuged with 16 000 g for 10 minutes at 4 °C. The separated upper and lower phases were collected and diluted in 3 mL of Ultima Gold^TM^ (Perkin Elmer, USA) and the average counts per minute (CPMA) were quantified in a Liquid Scintillation Analyzer (TRI-CARB 2100TR, Perkin Elmer life sciences, USA). The percentage of the AEA hydrolysis was calculated by dividing the CPMA of the upper phase, containing the ethanolamine-1-3H of the hydrolyzed ethanolamine-1-3H AEA with the CPMA sum of the upper and lower phase, followed by a multiplication with 100.

**Table S2** ANCOVAs controlled for SEX and AGE

|  | **Controls** | **NMPOU** | ***F*** | ***df1*** | ***df2*** | ***p*** | ***p_FDR_*** |
| --- | --- | --- | --- | --- | --- | --- | --- |
|  | (n=29) | (n=21) |  |  |  |  |  |
| AEA | 0.78 (0.2) | 0.92 (0.2) | 6.2 | 1 | 46 | 0.017 | 0.048 |
| OEA | 1.63 (0.5) | 2.07 (0.6) | 8.1 | 1 | 46 | 0.003 | 0.017 |
| PEA | 5.17 (1.1) | 6.26 (1.3) | 8.2 | 1 | 46 | 0.004 | 0.017 |
| LEA | 2.45 (0.8) | 2.70 (0.8) | 1.5 | 1 | 46 | 0.227 | 0.381 |
| SEA | 0.24 (0.1) | 0.26 (0.2) | 0.31 | 1 | 45 | 0.581 | 0.694 |
| 2-AG | 2.09 (0.9) | 2.27 (1.3) | 0.1 | 1 | 46 | 0.894 | 0.751 |
| 2-OG | 23.50 (14.4) | 31.02 (40.7) | 0.3 | 1 | 46 | 0.661 | 0.694 |
| SAG | 5.46 (1.6) | 6.31 (1.9) | 3.2 | 1 | 46 | 0.166 | 0.349 |
| 20:4PE | 67.62 (27.0) | 66.48 (29.2) | <.001 | 1 | 46 | 0.882 | 0.751 |
| AA | 154.8 (37.1) | 166.3 (48.0) | 2.6 | 1 | 46 | 0.347 | 0.486 |

Abbreviations: arachidonic acid (AA), anandamide (AEA), linoleoyl ethanolamide (LEA), oleoylethanolamide (OEA), palmitoylethanolamide (PEA), 1-Stearoyl-2-arachidonoyl-sn-glycerol (SAG), stearoyl ethanolamide (SEA), 2-arachidonylglycerol (2-AG), 2-oleoylglycerol (2-OG), 1,2-diarachidonoyl-sn-glycero-3-phosphoethanolamine (20:4PE).

**Table S3a** ANCOVAs controlled for SEX, AGE, and cannabis use within the last six months

|  | **Controls** | **NMPOU** | ***F*** | ***df1*** | ***df2*** | ***p*** | ***p_FDR_*** |
| --- | --- | --- | --- | --- | --- | --- | --- |
|  | (n=29) | (n=21) |  |  |  |  |  |
| AEA | 0.78 (0.2) | 0.92 (0.2) | 5.1 | 1 | 45 | 0.029 | 0.091 |
| OEA | 1.63 (0.5) | 2.07 (0.6) | 8.1 | 1 | 45 | 0.007 | 0.033 |
| PEA | 5.17 (1.1) | 6.26 (1.3) | 8.2 | 1 | 45 | 0.006 | 0.033 |
| LEA | 2.45 (0.8) | 2.70 (0.8) | 0.3 | 1 | 45 | 0.581 | 0.686 |
| SEA | 0.24 (0.1) | 0.26 (0.2) | 0.7 | 1 | 44 | 0.417 | 0.657 |
| 2-AG | 2.09 (0.9) | 2.27 (1.3) | 0.1 | 1 | 45 | 0.794 | 0.834 |
| 2-OG | 23.50 (14.4) | 31.02 (40.7) | 0.3 | 1 | 45 | 0.573 | 0.686 |
| SAG | 5.46 (1.6) | 6.31 (1.9) | 3.2 | 1 | 45 | 0.080 | 0.189 |
| 20:4PE | 67.62 (27.0) | 66.48 (29.2) | <.001 | 1 | 45 | 0.982 | 0.928 |
| AA | 154.8 (37.1) | 166.3 (48.0) | 2.6 | 1 | 45 | 0.116 | 0.219 |

**Table S3b** ANCOVAs controlled for SEX, AGE, and depression within the last six months

|  | **Controls** | **NMPOU** | ***F*** | ***df1*** | ***df2*** | ***p*** | ***p_FDR_*** |
| --- | --- | --- | --- | --- | --- | --- | --- |
|  | (n=29) | (n=21) |  |  |  |  |  |
| AEA | 0.78 (0.2) | 0.92 (0.2) | 5.7 | 1 | 45 | 0.021 | 0.074 |
| OEA | 1.63 (0.5) | 2.07 (0.6) | 6.6 | 1 | 45 | 0.013 | 0.068 |
| PEA | 5.17 (1.1) | 6.26 (1.3) | 6.7 | 1 | 45 | 0.013 | 0.068 |
| LEA | 2.45 (0.8) | 2.70 (0.8) | 0.9 | 1 | 45 | 0.341 | 0.639 |
| SEA | 0.24 (0.1) | 0.26 (0.2) | 0.04 | 1 | 44 | 0.851 | 0.894 |
| 2-AG | 2.09 (0.9) | 2.27 (1.3) | 0.1 | 1 | 45 | 0.741 | 0.894 |
| 2-OG | 23.50 (14.4) | 31.02 (40.7) | 0.1 | 1 | 45 | 0.781 | 0.894 |
| SAG | 5.46 (1.6) | 6.31 (1.9) | 0.8 | 1 | 45 | 0.365 | 0.639 |
| 20:4PE | 67.62 (27.0) | 66.48 (29.2) | 0.9 | 1 | 45 | 0.342 | 0.639 |
| AA | 154.8 (37.1) | 166.3 (48.0) | 0.2 | 1 | 45 | 0.662 | 0.894 |

**Table S3c** ANCOVAs controlled for SEX, AGE, and cigarettes used per week within the last six months

|  | **Controls** | **NMPOU** | ***F*** | ***df1*** | ***df2*** | ***p*** | ***p_FDR_*** |
| --- | --- | --- | --- | --- | --- | --- | --- |
|  | (n=29) | (n=21) |  |  |  |  |  |
| AEA | 0.78 (0.2) | 0.92 (0.2) | 4.6 | 1 | 45 | 0.038 | 0.133 |
| OEA | 1.63 (0.5) | 2.07 (0.6) | 7.0 | 1 | 45 | 0.011 | 0.089 |
| PEA | 5.17 (1.1) | 6.26 (1.3) | 6.1 | 1 | 45 | 0.017 | 0.089 |
| LEA | 2.45 (0.8) | 2.70 (0.8) | 0.8 | 1 | 45 | 0.370 | 0.648 |
| SEA | 0.24 (0.1) | 0.26 (0.2) | 1.9 | 1 | 44 | 0.183 | 0.384 |
| 2-AG | 2.09 (0.9) | 2.27 (1.3) | 0.2 | 1 | 45 | 0.683 | 0.719 |
| 2-OG | 23.50 (14.4) | 31.02 (40.7) | 0.2 | 1 | 45 | 0.685 | 0.719 |
| SAG | 5.46 (1.6) | 6.31 (1.9) | 0.5 | 1 | 45 | 0.489 | 0.719 |
| 20:4PE | 67.62 (27.0) | 66.48 (29.2) | 0.2 | 1 | 45 | 0.676 | 0.719 |
| AA | 154.8 (37.1) | 166.3 (48.0) | 3.1 | 1 | 45 | 0.087 | 0.228 |

Abbreviations: arachidonic acid (AA), anandamide (AEA), linoleoyl ethanolamide (LEA), oleoylethanolamide (OEA), palmitoylethanolamide (PEA), 1-Stearoyl-2-arachidonoyl-sn-glycerol (SAG), stearoyl ethanolamide (SEA), 2-arachidonylglycerol (2-AG), 2-oleoylglycerol (2-OG), 1,2-diarachidonoyl-sn-glycero-3-phosphoethanolamine (20:4PE).

**Table S4** Spearman correlations between *N*-acylethanolamines and alcohol, tobacco, and cannabis use within each user group

|  |  |  | AEA | OEA | PEA |
| --- | --- | --- | --- | --- | --- |
| **Alcohol use**^a^ | Last use in days | controls | -0.36 | -0.21 | -0.23 |
|  |  | NMPOU | -0.03 | 0.24 | -0.12 |
|  |  | over all | -0.16 | 0.02 | -0.03 |
|  | Gram/week | controls | 0.20 | 0.07 | 0.04 |
|  |  | NMPOU | -0.30 | -0.40 | -0.13 |
|  |  | over all | -0.12 | -0.20 | -0.20 |
|  | Use in years | controls | -0.12 | -0.06 | -0.15 |
|  |  | NMPOU | -0.04 | 0.06 | -0.13 |
|  |  | over all | -0.03 | 0.02 | -0.10 |
| **Nicotine use**^b^ | Last use in hours | controls | -0.07 | -0.24 | -0.13 |
|  |  | NMPOU | -0.26 | -0.32 | -0.30 |
|  |  | over all | -0.22 | -0.33 | -0.28 |
|  | Cigarettes/week | controls | 0.33 | **0.48** | 0.38 |
|  |  | NMPOU | 0.51 | 0.50 | 0.38 |
|  |  | over all | **0.48** | **0.56** | **0.52** |
|  | Use in years | controls | -0.45 | -0.30 | -0.34 |
|  |  | NMPOU | 0.20 | 0.29 | 0.16 |
|  |  | over all | -0.15 | -0.06 | -0.07 |
| **Cannabis use**^c^ | Last use in hours | controls | -0.47 | -0.38 | -0.05 |
|  |  | NMPOU | 0.30 | 0.17 | 0.16 |
|  |  | over all | -0.11 | -0.17 | -0.20 |
|  | Gram/week | controls | 0.26 | 0.20 | -0.08 |
|  |  | NMPOU | 0.10 | -0.09 | -0.02 |
|  |  | over all | 0.30 | 0.25 | 0.42 |
|  | Use in years | controls | 0.41 | 0.45 | -0.07 |
|  |  | NMPOU | 0.02 | -0.01 | 0.24 |
|  |  | over all | 0.24 | 0.18 | 0.16 |

^a^ Within alcohol users: controls n=27, NMPOU n=19, over all N=46

^b^ Within tobacco users: controls n=18, NMPOU n=15, over all N=33

^c^ Within cannabis users: controls n=9, NMPOU n=13, over all N=22

**Table S5a** Linear mixed model parameters with the fixed factor AEA, GROUP, and AEA*GROUP interactions controlled for AGE and SEX and their interactions.

Dependent variable: *estimated* *percentage ball received*

|  | Estimate | SE | CI | df | t | *p* |
| --- | --- | --- | --- | --- | --- | --- |
| (Intercept) | 33.86 | 32.44 | -31.29 – 99.01 | 50 | 1.04 | 0.302 |
| Group | -16.83 | 16.87 | -50.71 – 17.06 | 50 | -1.00 | 0.323 |
| AEA | -40.94 | 36.44 | -114.13 – 32.24 | 50 | -1.12 | 0.267 |
| **Group*AEA** | **42.06** | **13.33** | **15.29 – 68.83** | **50** | **3.16** | **0.003** |
| Sex | 0.66 | 12.41 | -24.26 – 25.58 | 50 | 0.05 | 0.958 |
| Age | 0.83 | 0.44 | -0.05 – 1.72 | 50 | 1.89 | 0.064 |
| AEA*Sex | 11.13 | 12.90 | -14.78 – 37.04 | 50 | 0.86 | 0.392 |
| AEA*Age | -1.00 | 0.63 | -2.26 – 0.26 | 50 | -1.60 | 0.117 |
| Group*Sex | -9.16 | 5.38 | -19.97 – 1.66 | 50 | -1.70 | 0.095 |
| Group*Age | -0.14 | 0.32 | -0.79 – 0.51 | 50 | -0.43 | 0.670 |

Dependent variable: *feeling included*

|  | Estimate | SE | CI | df | t | *p* |
| --- | --- | --- | --- | --- | --- | --- |
| (Intercept) | 9.31 | 5.37 | -1.47 – 20.01 | 50 | 1.74 | 0.089 |
| Group | -1.27 | 2.79 | -6.88 – 4.33 | 50 | -0.46 | 0.650 |
| AEA | -11.37 | 6.03 | -23.48 – 0.73 | 50 | -1.89 | 0.065 |
| **Group*AEA** | **5.86** | **2.20** | **1.43 – 10.29** | **50** | **2.66** | **0.011** |
| Sex | -1.98 | 2.05 | -6.10 – 2.14 | 50 | -0.96 | 0.340 |
| Age | 0.12 | 0.07 | -0.02 – 0.27 | 50 | 1.69 | 0.098 |
| AEA*Sex | 4.25 | 2.13 | -0.03 – 8.54 | 50 | 1.99 | 0.052 |
| AEA*Age | -0.06 | 0.10 | -0.27 – 0.15 | 50 | -0.60 | 0.554 |
| Group*Sex | -1.68 | 0.89 | -3.47 – 0.11 | 50 | -1.89 | 0.065 |
| Group*Age | -0.06 | 0.05 | -0.17 – 0.04 | 50 | -1.18 | 0.243 |

Dependent variable: *feeling excluded*

|  | Estimate | SE | CI | df | t | *p* |
| --- | --- | --- | --- | --- | --- | --- |
| (Intercept) | 0.97 | 7.10 | -13.29 – 15.24 | 50 | 0.14 | 0.892 |
| Group | 0.95 | 3.69 | -6.48 – 8.37 | 50 | 0.26 | 0.799 |
| AEA | 12.33 | 7.98 | -3.69 – 28.36 | 50 | 1.55 | 0.128 |
| Group*AEA | -4.97 | 2.92 | -10.83 – 0.90 | 50 | -1.70 | 0.095 |
| Sex | 2.61 | 2.72 | -2.85 – 8.07 | 50 | 0.96 | 0.341 |
| Age | -0.10 | 0.10 | -0.29 – 0.10 | 50 | -0.99 | 0.327 |
| AEA*Sex | -4.78 | 2.83 | -10.46 – 1.00 | 50 | -1.69 | 0.097 |
| AEA*Age | -0.02 | 0.14 | -0.30 – 0.26 | 50 | -0.14 | 0.891 |
| Group*Sex | 1.10 | 1.18 | -1.27 – 3.47 | 50 | 0.93 | 0.357 |
| Group*Age | 0.07 | 0.07 | -0.07 – 0.21 | 50 | 0.98 | 0.332 |

Dependent variable: *change score positive affect (PA)*

|  | Estimate | SE | CI | df | t | *p* |
| --- | --- | --- | --- | --- | --- | --- |
| (Intercept) | -4.25 | 2.58 | -9.44 – 0.95 | 50 | -1.64 | 0.107 |
| Group | 2.34 | 1.34 | -0.36 – 5.04 | 50 | 1.74 | 0.088 |
| AEA | 2.99 | 2.90 | -2.85 – 8.82 | 50 | 1.03 | 0.309 |
| Group*AEA | -1.36 | 1.06 | -3.49 – 0.78 | 50 | -1.28 | 0.207 |
| Sex | -0.10 | 0.99 | -2.08 – 1.90 | 50 | -0.10 | 0.922 |
| Age | 0.07 | 0.04 | 0.004 – 0.14 | 50 | 2.11 | 0.040 |
| AEA*Sex | -0.38 | 1.03 | -2.44 – 1.69 | 50 | -0.37 | 0.716 |
| AEA*Age | -0.01 | 0.05 | -0.11 – 0.09 | 50 | -0.13 | 0.894 |
| Group*Sex | 0.14 | 0.43 | -0.73 – 0.10 | 50 | 0.32 | 0.753 |
| Group*Age | -0.04 | 0.03 | -0.09 – 0.01 | 50 | -1.58 | 0.120 |

Dependent variable: *change score negative affect (NA)*

|  | Estimate | SE | CI | df | t | *p* |
| --- | --- | --- | --- | --- | --- | --- |
| (Intercept) | 1.86 | 1.14 | -0.44 – 4.15 | 50 | 1.62 | 0.111 |
| Group | -1.09 | 0.60 | -2.29 – 0.10 | 50 | -1.84 | 0.073 |
| AEA | -0.55 | 1.28 | -3.13 – 2.03 | 50 | -0.43 | 0.672 |
| Group*AEA | 0.07 | 0.47 | -0.87 – 1.01 | 50 | 0.15 | 0.881 |
| Sex | -0.03 | 0.44 | -0.91 – 0.85 | 50 | -0.07 | 0.947 |
| **Age** | **-0.05** | **0.02** | **-0.08 – -0.01** | **50** | **-2.89** | **0.006** |
| AEA*Sex | -0.18 | 0.46 | -1.09 – 0.73 | 50 | -0.39 | 0.702 |
| AEA*Age | 0.01 | 0.02 | -0.03 – 0.06 | 50 | 0.49 | 0.629 |
| Group*Sex | 0.21 | 0.19 | -0.17 – 0.60 | 50 | 1.13 | 0.265 |
| **Group*Age** | **0.02** | **0.01** | **>0.01 – 0.05** | **50** | **2.03** | **0.048** |

**Table S5b** Linear mixed model parameters with the fixed factor OEA, GROUP, and OEA*GROUP interactions controlled for AGE and SEX and their interactions.

Dependent variable: *estimated* *percentage ball received*

|  | Estimate | SE | CI | df | t | *p* |
| --- | --- | --- | --- | --- | --- | --- |
| (Intercept) | 26.13 | 29.32 | -32.77 – 85.03 | 50 | 0.89 | 0.377 |
| Group | -15.54 | 16.22 | -48.13 – 17.04 | 50 | -0.96 | 0.343 |
| OEA | -11.54 | 13.76 | -39.18 – 16.09 | 50 | -0.84 | 0.406 |
| **Group*OEA** | **16.16** | **4.69** | **6.74 – 25.57** | **50** | **3.45** | **0.001** |
| Sex | 3.56 | 11.10 | -18.73 – 25.85 | 50 | 0.32 | 0.750 |
| Age | 0.78 | 0.40 | -0.02 – 1.58 | 50 | 1.97 | 0.055 |
| OEA*Sex | 1.09 | 4.89 | -8.74 – 10.92 | 50 | 0.22 | 0.825 |
| OEA*Age | -0.41 | 0.24 | -0.88 – 0.07 | 50 | -1.73 | 0.090 |
| Group*Sex | -6.20 | 5.18 | -16.60 – 4.19 | 50 | -1.20 | 0.237 |
| Group*Age | -0.14 | 0.31 | -0.78 – 0.49 | 50 | -0.46 | 0.649 |

Dependent variable: *feeling included*

|  | Estimate | SE | CI | df | t | *p* |
| --- | --- | --- | --- | --- | --- | --- |
| (Intercept) | 8.76 | 5.09 | -1.47 – 18.99 | 50 | 1.72 | 0.092 |
| Group | 0.68 | 2.82 | -4.98 – 6.34 | 50 | 0.24 | 0.811 |
| **OEA** | **-4.97** | **2.39** | **-9.77 – -0.17** | **50** | **-2.08** | **0.043** |
| **Group*OEA** | **1.66** | **0.81** | **0.02 – 3.29** | **50** | **2.03** | **0.047** |
| Sex | -1.97 | 1.93 | -5.84 – 1.90 | 50 | -1.02 | 0.312 |
| Age | 0.09 | 0.07 | -0.05 – 0.23 | 50 | 1.30 | 0.200 |
| OEA*Sex | 1.65 | 0.85 | -0.06 – 3.35 | 50 | 1.94 | 0.058 |
| OEA*Age | 0.01 | 0.04 | -0.07 – 0.10 | 50 | 0.31 | 0.759 |
| Group*Sex | -1.30 | 0.90 | -3.11 – 0.51 | 50 | -1.45 | 0.155 |
| Group*Age | -0.09 | 0.06 | -0.20 – 0.02 | 50 | -1.65 | 0.106 |

Dependent variable: *feeling excluded*

|  | Estimate | SE | CI | df | t | *p* |
| --- | --- | --- | --- | --- | --- | --- |
| (Intercept) | 4.51 | 6.83 | -9.21 – 18.23 | 50 | 0.66 | 0.512 |
| Group | -0.33 | 3.78 | -7.92 – 7.26 | 50 | -0.09 | 0.930 |
| OEA | 3.28 | 3.21 | -3.16 – 9.72 | 50 | 1.02 | 0.311 |
| Group*OEA | -1.36 | 1.09 | -3.55 – 0.83 | 50 | -1.25 | 0.219 |
| Sex | 1.00 | 2.59 | -4.19 – 6.19 | 50 | 0.39 | 0.701 |
| Age | -0.08 | 0.09 | -0.26 – 0.11 | 50 | -0.84 | 0.406 |
| OEA*Sex | -1.19 | 1.14 | -3.48 – 1.10 | 50 | -1.05 | 0.300 |
| OEA*Age | -0.01 | 0.06 | -0.12 – 0.10 | 50 | -0.14 | 0.887 |
| Group*Sex | 0.93 | 1.21 | -1.49 – 3.35 | 50 | 0.77 | 0.444 |
| Group*Age | 0.06 | 0.07 | -0.09 – 0.21 | 50 | 0.83 | 0.409 |

Dependent variable: *change score positive affect (PA)*

|  | Estimate | SE | CI | df | t | *p* |
| --- | --- | --- | --- | --- | --- | --- |
| (Intercept) | -4.06 | 2.40 | -8.89 – 0.77 | 50 | -1.69 | 0.097 |
| Group | 2.45 | 1.33 | -0.22 – 5.13 | 50 | 1.85 | 0.071 |
| OEA | 1.05 | 1.13 | -1.22 – 3.32 | 50 | 0.93 | 0.356 |
| Group*OEA | -0.56 | 0.38 | -1.34 – 0.21 | 50 | -1.47 | 0.149 |
| Sex | 0.03 | 0.91 | -1.79 – 1.86 | 50 | 0.04 | 0.971 |
| Age | 0.07 | 0.03 | >0.00 – 0.13 | 50 | 2.02 | 0.049 |
| OEA*Sex | -0.14 | 0.40 | -0.95 – 0.67 | 50 | -0.35 | 0.728 |
| OEA*Age | 0.00 | 0.02 | -0.04 – 0.04 | 50 | 0.22 | 0.830 |
| Group*Sex | 0.05 | 0.42 | -0.80 – 0.90 | 50 | 0.12 | 0.908 |
| Group*Age | -0.04 | 0.03 | -0.10 – 0.01 | 50 | -1.73 | 0.090 |

Dependent variable: *change score negative affect (NA)*

|  | Estimate | SE | CI | df | t | *p* |
| --- | --- | --- | --- | --- | --- | --- |
| (Intercept) | 1.66 | 1.09 | -0.54 – 3.86 | 50 | 1.52 | 0.135 |
| Group | -1.07 | 0.61 | -2.29 – 0.14 | 50 | -1.78 | 0.082 |
| OEA | -0.17 | 0.51 | -1.20 – 0.86 | 50 | -0.33 | 0.744 |
| Group*OEA | 0.05 | 0.18 | -0.30 – 0.40 | 50 | 0.29 | 0.772 |
| Sex | -0.10 | 0.41 | -0.93 – 0.73 | 50 | -0.24 | 0.809 |
| **Age** | **-0.04** | **0.02** | **-0.07 – -0.01** | **50** | **-2.54** | **0.014** |
| OEA*Sex | -0.05 | 0.18 | -0.42 – 0.31 | 50 | -0.30 | 0.767 |
| OEA*Age | >0.00 | 0.01 | -0.02 – 0.02 | 50 | 0.31 | 0.756 |
| Group*Sex | 0.21 | 0.19 | -0.18 – 0.60 | 50 | 1.08 | 0.285 |
| Group*Age | 0.02 | 0.01 | >0.00 – 0.05 | 50 | 1.83 | 0.074 |

**Table S5c** Linear mixed model parameters with the fixed factor PEA, GROUP, and PEA*GROUP interactions controlled for AGE and SEX and their interactions.

Dependent variable: *estimated* *percentage ball received*

|  | Estimate | SE | CI | df | t | *p* |
| --- | --- | --- | --- | --- | --- | --- |
| (Intercept) | -9.58 | 38.04 | -85.98 – 66.83 | 50 | -0.25 | 0.802 |
| Group | 9.77 | 17.82 | -26.02 – 45.57 | 50 | 0.55 | 0.586 |
| PEA | -0.49 | 6.30 | -13.14 – 12.16 | 50 | -0.08 | 0.938 |
| Group*PEA | 2.51 | 2.07 | -1.64 – 6.66 | 50 | 1.21 | 0.230 |
| Sex | 13.19 | 14.93 | -16.80 – 43.18 | 50 | 0.88 | 0.381 |
| Age | 0.67 | 0.65 | -0.63 – 1.97 | 50 | 1.04 | 0.305 |
| PEA*Sex | -1.25 | 2.31 | -5.88 – 3.39 | 50 | -0.54 | 0.591 |
| PEA*Age | >0.00 | 0.14 | -0.29 – 0.29 | 50 | -0.01 | 0.994 |
| Group*Sex | -6.10 | 5.88 | -17.91 – 5.72 | 50 | -1.04 | 0.305 |
| Group*Age | -0.50 | 0.33 | -1.16 – 0.16 | 50 | -1.51 | 0.137 |

Dependent variable: *feeling included*

|  | Estimate | SE | CI | df | t | *p* |
| --- | --- | --- | --- | --- | --- | --- |
| (Intercept) | -0.92 | 6.33 | -13.63 – 11.79 | 50 | -0.15 | 0.885 |
| Group | 3.81 | 2.97 | -2.14 – 9.77 | 50 | 1.29 | 0.204 |
| PEA | -0.08 | 1.05 | -2.18 – 2.02 | 50 | -0.08 | 0.939 |
| Group*PEA | 0.08 | 0.34 | -0.61 – 0.77 | 50 | 0.22 | 0.825 |
| Sex | 1.57 | 2.48 | -3.42 – 6.56 | 50 | 0.63 | 0.529 |
| Age | 0.05 | 0.11 | -0.16 – 0.27 | 50 | 0.48 | 0.630 |
| PEA*Sex | -0.09 | 0.38 | -0.86 – 0.69 | 50 | -0.22 | 0.827 |
| PEA*Age | 0.02 | 0.02 | -0.03 – 0.06 | 50 | 0.63 | 0.529 |
| Group*Sex | -1.26 | 0.98 | -3.23 – 0.71 | 50 | -1.29 | 0.204 |
| Group*Age | -0.10 | 0.06 | -0.21 – 0.01 | 50 | -1.85 | 0.071 |

Dependent variable: *feeling excluded*

|  | Estimate | SE | CI | df | t | *p* |
| --- | --- | --- | --- | --- | --- | --- |
| (Intercept) | 6.77 | 8.37 | -10.03 – 23.57 | 50 | 0.81 | 0.422 |
| Group | -1.30 | 3.92 | -9.17 – 6.58 | 50 | -0.33 | 0.743 |
| PEA | 0.55 | 1.39 | -2.23 – 3.33 | 50 | 0.40 | 0.692 |
| Group*PEA | -0.16 | 0.46 | -1.07 – 0.75 | 50 | -0.35 | 0.725 |
| Sex | 0.40 | 3.28 | -6.20 – 6.99 | 50 | 0.12 | 0.904 |
| Age | -0.04 | 0.14 | -0.33 – 0.25 | 50 | -0.28 | 0.782 |
| PEA*Sex | -0.22 | 0.51 | -1.24 – 0.08 | 50 | -0.43 | 0.672 |
| PEA*Age | -0.01 | 0.03 | -0.07 – 0.06 | 50 | -0.23 | 0.823 |
| Group*Sex | 0.61 | 1.29 | -1.99 – 3.20 | 50 | 0.47 | 0.642 |
| Group*Age | 0.05 | 0.07 | -0.10 – 0.20 | 50 | 0.68 | 0.503 |

Dependent variable: *change score positive affect (PA)*

|  | Estimate | SE | CI | df | t | *p* |
| --- | --- | --- | --- | --- | --- | --- |
| (Intercept) | -6.20 | 2.71 | -11.63 – -0.76 | 50 | -2.29 | 0.026 |
| Group | 1.89 | 1.27 | -0.66 – 4.44 | 50 | 1.49 | 0.142 |
| **PEA** | **0.95** | **0.45** | **0.05 – 1.85** | **50** | **2.13** | **0.038** |
| Group*PEA | -0.24 | 0.15 | -0.53 – 0.06 | 50 | -1.61 | 0.113 |
| Sex | 0.07 | 1.06 | -2.06 – 2.21 | 50 | 0.07 | 0.947 |
| **Age** | **0.12** | **0.05** | **0.03 – 0.22** | **50** | **2.66** | **0.010** |
| PEA*Sex | -0.05 | 0.16 | -0.38 – 0.28 | 50 | -0.30 | 0.766 |
| PEA*Age | -0.02 | 0.01 | -0.04 – 0.003 | 50 | -1.71 | 0.094 |
| Group*Sex | 0.08 | 0.42 | -0.77 – 0.92 | 50 | 0.18 | 0.857 |
| Group*Age | -0.02 | 0.02 | -0.06 – 0.03 | 50 | -0.63 | 0.533 |

Dependent variable: *change score negative affect (NA)*

|  | Estimate | SE | CI | df | t | *p* |
| --- | --- | --- | --- | --- | --- | --- |
| (Intercept) | 2.79 | 1.21 | 0.37 – 5.21 | 50 | 2.31 | 0.025 |
| **Group** | **-1.22** | **0.57** | **-2.35 – -0.08** | **50** | **-2.15** | **0.036** |
| PEA | -0.33 | 0.20 | -0.73 – 0.07 | 50 | -1.65 | 0.105 |
| Group*PEA | 0.10 | 0.07 | -0.03 – 0.23 | 50 | 1.50 | 0.140 |
| Sex | -0.02 | 0.47 | -0.97 – 0.93 | 50 | -0.04 | 0.967 |
| **Age** | **-0.06** | **0.02** | **-0.10 – -0.02** | **50** | **-2.82** | **0.007** |
| PEA*Sex | -0.04 | 0.07 | -0.18 – 0.11 | 50 | -0.49 | 0.624 |
| PEA*Age | 0.01 | 0.01 | -0.002 – 0.02 | 50 | 1.59 | 0.119 |
| Group*Sex | 0.19 | 0.19 | -0.19 – 0.56 | 50 | 1.01 | 0.319 |
| Group*Age | 0.01 | 0.01 | -0.01 – 0.03 | 50 | 1.11 | 0.271 |

Abbreviations: anandamide (AEA), oleoylethanolamide (OEA), palmitoylethanolamide (PEA)

**Table S6a** Linear mixed model parameters with the fixed factor AEA, GROUP, and AEA*GROUP interactions controlled for AGE, SEX, their interactions, and cannabis use.

Dependent variable: *estimated* *percentage ball received*

|  | Estimate | SE | CI | df | t | *p* |
| --- | --- | --- | --- | --- | --- | --- |
| (Intercept) | 36.79 | 32.49 | -28.46 – 102.05 | 50 | 1.13 | 0.263 |
| Group | -17.53 | 16.80 | -51.28 – 16.21 | 50 | -1.04 | 0.302 |
| AEA | -41.11 | 36.23 | -113.89 – 31.66 | 50 | -1.14 | 0.262 |
| **Group*AEA** | 42.40 | 13.26 | **15.77 –**  **69.03** | **50** | **3.20** | **0.002** |
| Sex | 1.10 | 12.35 | -23.71 – 25.90 | 50 | 0.09 | 0.930 |
| Age | 0.85 | 0.44 | -0.03 – 1.73 | 50 | 1.94 | 0.058 |
| Cannabis use | -1.78 | 2.36 | -6.52 – 2.96 | 50 | -0.75 | 0.455 |
| AEA*Sex | 10.54 | 12.85 | -15.27 – 36.35 | 50 | 0.82 | 0.416 |
| AEA*Age | -0.98 | 0.62 | -2.24 – 0.27 | 50 | -1.58 | 0.121 |
| Group*Sex | -9.22 | 5.35 | -19.98 – 1.53 | 50 | -1.72 | 0.091 |
| Group*Age | -0.14 | 0.32 | -0.79 – 0.51 | 50 | -0.44 | 0.665 |

Dependent variable: *feeling included*

|  | Estimate | SE | CI | df | t | *p* |
| --- | --- | --- | --- | --- | --- | --- |
| (Intercept) | 10.35 | 5.26 | -0.22 – 20.92 | 50 | 1.97 | 0.055 |
| Group | -1.52 | 2.72 | -6.99 – 3.95 | 50 | -0.56 | 0.579 |
| AEA | -11.43 | 5.87 | -23.22 – 0.36 | 50 | -1.95 | 0.057 |
| **Group*AEA** | 5.98 | 2.15 | **1.66 – 10.29** | **50** | **2.78** | **0.008** |
| Sex | -1.82 | 2.00 | -5.84 – 2.20 | 50 | -0.91 | 0.367 |
| Age | 0.13 | 0.07 | -0.01 – 0.27 | 50 | 1.82 | 0.076 |
| Cannabis use | -0.63 | 0.38 | -1.40 – 0.14 | 50 | -1.65 | 0.106 |
| AEA*Sex | 4.04 | 2.08 | -0.14 – 8.22 | 50 | 1.94 | 0.058 |
| AEA*Age | -0.06 | 0.10 | -0.26 – 0.15 | 50 | -0.55 | 0.585 |
| Group*Sex | -1.70 | 0.87 | -3.45 – 0.04 | 50 | -1.96 | 0.055 |
| Group*Age | -0.06 | 0.05 | -0.17 – 0.04 | 50 | -1.22 | 0.228 |

Dependent variable: *feeling excluded*

|  | Estimate | SE | CI | df | t | *p* |
| --- | --- | --- | --- | --- | --- | --- |
| (Intercept) | 0.49 | 7.13 | -13.83 – 14.82 | 50 | 0.07 | 0.945 |
| Group | 1.06 | 3.69 | -6.35 – 8.47 | 50 | 0.29 | 0.775 |
| AEA | 12.36 | 7.96 | -3.62 – 28.34 | 50 | 1.55 | 0.126 |
| Group*AEA | -5.02 | 2.91 | -10.87 – 0.83 | 50 | -1.73 | 0.091 |
| Sex | 2.54 | 2.71 | -2.91 – 7.99 | 50 | 0.94 | 0.354 |
| Age | -0.10 | 0.10 | -0.29 – 0.10 | 50 | -1.02 | 0.312 |
| Cannabis use | 0.29 | 0.52 | -0.75 – 1.33 | 50 | 0.56 | 0.577 |
| AEA*Sex | -4.69 | 2.82 | -10.35 – 0.98 | 50 | -1.66 | 0.103 |
| AEA*Age | -0.02 | 0.14 | -0.30 – 0.25 | 50 | -0.16 | 0.874 |
| Group*Sex | 1.11 | 1.18 | -1.25 – 3.47 | 50 | 0.94 | 0.351 |
| Group*Age | 0.07 | 0.07 | -0.07 – 0.21 | 50 | 0.99 | 0.329 |

Dependent variable: *change score positive affect (PA)*

|  | Estimate | SE | CI | df | t | *p* |
| --- | --- | --- | --- | --- | --- | --- |
| (Intercept) | -4.30 | 2.60 | -9.53 – 0.93 | 50 | -1.65 | 0.105 |
| Group | 2.35 | 1.35 | -0.35 – 5.06 | 50 | 1.75 | 0.087 |
| AEA | 2.99 | 2.90 | -2.84 – 8.82 | 50 | 1.03 | 0.308 |
| Group*AEA | -1.36 | 1.06 | -3.50 – 0.77 | 50 | -1.28 | 0.205 |
| Sex | -0.11 | 0.99 | -2.09 – 1.88 | 50 | -0.11 | 0.916 |
| Age | 0.07 | 0.04 | 0.003 – 0.14 | 50 | 2.10 | 0.041 |
| Cannabis use | 0.03 | 0.19 | -0.35 – 0.41 | 50 | 0.17 | 0.865 |
| AEA*Sex | -0.37 | 1.03 | -2.43 – 1.70 | 50 | -0.36 | 0.724 |
| AEA*Age | -0.01 | 0.05 | -0.11 – 0.09 | 50 | -0.14 | 0.889 |
| Group*Sex | 0.14 | 0.43 | -0.72 – 1.00 | 50 | 0.32 | 0.751 |
| Group*Age | -0.04 | 0.03 | -0.09 – 0.01 | 50 | -1.58 | 0.120 |

Dependent variable: *change score negative affect (NA)*

|  | Estimate | SE | CI | df | t | *p* |
| --- | --- | --- | --- | --- | --- | --- |
| (Intercept) | 1.73 | 1.14 | -0.56 – 4.03 | 50 | 1.52 | 0.135 |
| Group | -1.06 | 0.59 | -2.25 – 0.13 | 50 | -1.80 | 0.078 |
| AEA | -0.54 | 1.27 | -3.10 – 2.02 | 50 | -0.42 | 0.674 |
| Group*AEA | 0.06 | 0.47 | -0.88 – 0.99 | 50 | 0.12 | 0.904 |
| Sex | -0.05 | 0.43 | -0.92 – 0.83 | 50 | -0.11 | 0.914 |
| **Age** | **-0.05** | **0.02** | **-0.08 – -0.02** | **50** | **-2.95** | **0.005** |
| Cannabis use | 0.08 | 0.08 | -0.09 – 0.24 | 50 | 0.90 | 0.372 |
| AEA*Sex | -0.15 | 0.45 | -1.06 – 0.76 | 50 | -0.33 | 0.741 |
| AEA*Age | 0.01 | 0.02 | -0.03 – 0.05 | 50 | 0.46 | 0.650 |
| Group*Sex | 0.22 | 0.19 | -0.16 – 0.60 | 50 | 1.15 | 0.255 |
| **Group*Age** | **0.02** | **0.01** | **>0.01 – 0.05** | **50** | **2.05** | **0.046** |

**Table S6b** Linear mixed model parameters with the fixed factor OEA, GROUP, and OEA*GROUP interactions controlled for AGE, SEX, their interactions, and cannabis use.

Dependent variable: *estimated* *percentage ball received*

|  | Estimate | SE | CI | df | t | *p* |
| --- | --- | --- | --- | --- | --- | --- |
| (Intercept) | 29.63 | 29.30 | -29.23 – 88.49 | 50 | 1.01 | 0.317 |
| Group | -16.63 | 16.12 | -49.01 – 15.75 | 50 | -1.03 | 0.307 |
| OEA | -11.57 | 13.64 | -38.96 – 15.82 | 50 | -0.85 | 0.4 |
| **Group*OEA** | **16.48** | **4.66** | **7.12 – 25.84** | **50** | **3.54** | **<.001** |
| Sex | 4.15 | 11.02 | -17.98 – 26.28 | 50 | 0.38 | 0.708 |
| Age | 0.81 | 0.40 | 0.02 – 1.60 | 50 | 2.05 | 0.046 |
| Cannabis use | -2.17 | 2.30 | -6.79 – 2.46 | 50 | -0.94 | 0.351 |
| OEA*Sex | 0.67 | 4.87 | -9.12 – 10.45 | 50 | 0.14 | 0.891 |
| OEA*Age | -0.40 | 0.23 | -0.87 – 0.07 | 50 | -1.72 | 0.093 |
| Group*Sex | -6.26 | 5.13 | -16.56 – 4.05 | 50 | -1.22 | 0.228 |
| Group*Age | -0.15 | 0.31 | -0.77 – 0.48 | 50 | -0.47 | 0.642 |

Dependent variable: *feeling included*

|  | Estimate | SE | CI | df | t | *p* |
| --- | --- | --- | --- | --- | --- | --- |
| (Intercept) | 9.83 | 4.99 | -0.20 – 19.86 | 50 | 1.97 | 0.054 |
| Group | 0.34 | 2.75 | -5.17 –5.86 | 50 | 0.13 | 0.901 |
| **OEA** | **-4.98** | **2.32** | **-9.64 – -0.31** | **50** | **-2.14** | **0.037** |
| **Group*OEA** | **1.75** | **0.79** | **0.16 – 3.35** | **50** | **2.21** | **0.032** |
| Sex | -1.79 | 1.88 | -5.56 – 1.98 | 50 | -0.95 | 0.346 |
| Age | 0.10 | 0.07 | -0.04 – 0.23 | 50 | 1.46 | 0.151 |
| Cannabis use | -0.66 | 0.39 | -1.45 – 0.12 |  | -1.69 | 0.096 |
| OEA*Sex | 1.52 | 0.83 | -0.15 – 3.18 | 50 | 1.83 | 0.073 |
| OEA*Age | 0.02 | 0.04 | -0.07 – 0.09 | 50 | 0.37 | 0.715 |
| Group*Sex | -1.32 | 0.87 | -3.07 – 0.44 | 50 | -1.51 | 0.138 |
| Group*Age | -0.09 | 0.05 | -0.20 – 0.02 | 50 | -1.70 | 0.095 |

Dependent variable: *feeling excluded*

|  | Estimate | SE | CI | df | t | *p* |
| --- | --- | --- | --- | --- | --- | --- |
| (Intercept) | 3.98 | 6.86 | -9.80 – 17.76 | 50 | 0.58 | 0.564 |
| Group | -0.17 | 3.77 | -7.75 – 7.41 | 50 | -0.04 | 0.965 |
| OEA | 3.29 | 3.19 | -3.13 – 9.70 | 50 | 1.03 | 0.308 |
| Group*OEA | -1.41 | 1.09 | -3.60 – 0.78 | 50 | -1.29 | 0.202 |
| Sex | 0.91 | 2.58 | -4.27 – 6.09 | 50 | 0.35 | 0.727 |
| Age | -0.08 | 0.09 | -0.27 – 0.10 | 50 | -0.88 | 0.381 |
| Cannabis use | 0.33 | 0.54 | -0.75 – 1.41 | 50 | 0.61 | 0.543 |
| OEA*Sex | -1.13 | 1.14 | -3.42 – 1.16 | 50 | -0.99 | 0.327 |
| OEA*Age | -0.01 | 0.06 | -0.12 – 0.10 | 50 | -0.16 | 0.873 |
| Group*Sex | 0.94 | 1.20 | -1.47 – 3.35 | 50 | 0.78 | 0.438 |
| Group*Age | 0.06 | 0.07 | -0.09 – 0.21 | 50 | 0.84 | 0.406 |

Dependent variable: *change score positive affect (PA)*

|  | Estimate | SE | CI | df | t | *p* |
| --- | --- | --- | --- | --- | --- | --- |
| (Intercept) | -4.11 | 2.42 | -8.98 – 0.76 | 50 | -1.70 | 0.096 |
| Group | 2.47 | 1.33 | -0.21 – 5.15 | 50 | 1.85 | 0.070 |
| OEA | 1.05 | 1.13 | -1.22 – 3.32 | 50 | 0.93 | 0.356 |
| Group*OEA | -0.57 | 0.39 | -1.34 – 0.21 | 50 | -1.48 | 0.147 |
| Sex | 0.03 | 0.91 | -1.80 – 1.86 | 50 | 0.03 | 0.978 |
| Age | 0.07 | 0.03 | >0.00 – 0.13 | 50 | 2.00 | 0.051 |
| Cannabis use | 0.03 | 0.19 | -0.35 – 0.41 | 50 | 0.16 | 0.878 |
| OEA*Sex | -0.14 | 0.40 | -0.94 – 0.67 | 50 | -0.33 | 0.739 |
| OEA*Age | 0.00 | 0.02 | -0.04 – 0.04 | 50 | 0.21 | 0.834 |
| Group*Sex | 0.05 | 0.42 | -0.80 – 0.90 | 50 | 0.12 | 0.907 |
| Group*Age | -0.04 | 0.03 | -0.10 – 0.01 | 50 | -1.73 | 0.090 |

Dependent variable: *change score negative affect (NA)*

|  | Estimate | SE | CI | df | t | *p* |
| --- | --- | --- | --- | --- | --- | --- |
| (Intercept) | 1.53 | 1.09 | -0.66 – 3.73 | 50 | 1.40 | 0.167 |
| Group | -1.03 | 0.60 | -2.24 – 0.17 | 50 | -1.72 | 0.092 |
| OEA | -0.17 | 0.51 | -1.19 – 0.85 | 50 | -0.33 | 0.744 |
| Group*OEA | 0.04 | 0.17 | -0.31 – 0.39 | 50 | 0.22 | 0.823 |
| Sex | -0.12 | 0.41 | -0.95 – 0.70 | 50 | -0.30 | 0.767 |
| **Age** | **-0.04** | **0.02** | **-0.07 – -0.01** | **50** | **-2.62** | **0.012** |
| Cannabis use | 0.08 | 0.09 | -0.09 – 0.25 | 50 | 0.92 | 0.361 |
| OEA*Sex | -0.04 | 0.18 | -0.40 – 0.33 | 50 | -0.21 | 0.831 |
| OEA*Age | 0.00 | 0.01 | -0.02 – 0.02 | 50 | 0.29 | 0.775 |
| Group*Sex | 0.21 | 0.19 | -0.17 – 0.60 | 50 | 1.10 | 0.276 |
| Group*Age | 0.02 | 0.01 | >0.00 – 0.05 | 50 | 1.85 | 0.070 |

**Table S6c** Linear mixed model parameters with the fixed factor PEA, GROUP, and PEA*GROUP interactions controlled for AGE, SEX, their interactions, and cannabis use.

Dependent variable: *estimated* *percentage ball received*

|  | Estimate | SE | CI | df | t | *p* |
| --- | --- | --- | --- | --- | --- | --- |
| (Intercept) | -6.64 | 37.84 | -82.64– 69.35 | 50 | -0.18 | 0.861 |
| Group | 9.50 | 17.67 | -25.99 – 44.99 | 50 | 0.54 | 0.593 |
| PEA | -0.58 | 6.24 | -13.11 – 11.96 | 50 | -0.09 | 0.927 |
| Group*PEA | 2.65 | 2.06 | -1.48 – 6.78 | 50 | 1.29 | 0.204 |
| Sex | 15.08 | 14.94 | -14.92 – 45.08 | 50 | 1.01 | 0.317 |
| Age | 0.65 | 0.64 | -0.64 – 1.94 | 50 | 1.02 | 0.314 |
| Cannabis use | -2.42 | 2.58 | -7.60 – 2.76 | 50 | -0.94 | 0.352 |
| PEA*Sex | -1.58 | 2.32 | -6.23 – 3.07 | 50 | -0.68 | 0.497 |
| PEA*Age | 0.02 | 0.14 | -0.27 – 0.31 | 50 | 0.12 | 0.906 |
| Group*Sex | -6.38 | 5.84 | -18.11 – 5.34 | 50 | -1.09 | 0.280 |
| Group*Age | -0.53 | 0.33 | -1.19 – 0.14 | 50 | -1.60 | 0.116 |

Dependent variable: *feeling included*

|  | Estimate | SE | CI | df | t | *p* |
| --- | --- | --- | --- | --- | --- | --- |
| (Intercept) | 0.02 | 6.14 | -12.32 – 12.36 | 50 | 0.003 | 0.997 |
| Group | 3.73 | 2.87 | -2.04 – 9.49 | 50 | 1.30 | 0.200 |
| PEA | -0.11 | 1.01 | -2.14 – 1.93 | 50 | -0.11 | 0.916 |
| Group*PEA | 0.12 | 0.33 | -0.55 – 0.79 | 50 | 0.36 | 0.720 |
| Sex | 2.18 | 2.43 | -2.69 – 7.05 | 50 | 0.90 | 0.373 |
| Age | 0.05 | 0.10 | -0.16 – 0.26 | 50 | 0.45 | 0.658 |
| Cannabis use | -0.78 | 0.42 | -1.62 – 0.07 | 50 | -1.85 | 0.070 |
| PEA*Sex | -0.19 | 0.38 | -0.95 – 0.56 | 50 | -0.51 | 0.613 |
| PEA*Age | 0.02 | 0.02 | -0.03 – 0.07 | 50 | 0.90 | 0.374 |
| Group*Sex | -1.35 | 0.95 | -3.26 – 0.55 | 50 | -1.43 | 0.160 |
| **Group*Age** | **-0.11** | **0.05** | **-0.22 – <0.01** | **50** | **-2.06** | **0.045** |

Dependent variable: *feeling excluded*

|  | Estimate | SE | CI | df | t | *p* |
| --- | --- | --- | --- | --- | --- | --- |
| (Intercept) | 6.34 | 8.36 | -10.46 – 23.13 | 50 | 0.76 | 0.452 |
| Group | -1.25 | 3.91 | -9.10 – 6.59 | 50 | -0.32 | 0.749 |
| PEA | 0.57 | 1.38 | -2.21 – 3.34 | 50 | 0.41 | 0.684 |
| Group*PEA | -0.18 | 0.45 | -1.09 – 0.73 | 50 | -0.40 | 0.692 |
| Sex | 0.12 | 3.30 | -6.51 – 6.75 | 50 | 0.04 | 0.971 |
| Age | -0.04 | 0.14 | -0.32 – 0.25 | 50 | -0.26 | 0.795 |
| Cannabis use | 0.36 | 0.57 | -0.79 – 1.50 | 50 | 0.63 | 0.535 |
| PEA*Sex | -0.17 | 0.51 | -1.19 – 0.86 | 50 | -0.33 | 0.746 |
| PEA*Age | -0.01 | 0.03 | -0.07 – 0.05 | 50 | -0.31 | 0.760 |
| Group*Sex | 0.65 | 1.29 | -1.94 – 3.24 | 50 | 0.50 | 0.618 |
| Group*Age | 0.05 | 0.07 | -0.09 – 0.20 | 50 | 0.73 | 0.471 |

Dependent variable: *change score positive affect (PA)*

|  | Estimate | SE | CI | df | t | *p* |
| --- | --- | --- | --- | --- | --- | --- |
| (Intercept) | -6.29 | 2.71 | -11.73 – -0.84 | 50 | -2.32 | 0.025 |
| Group | 1.90 | 1.27 | -0.65 – 4.44 | 50 | 1.50 | 0.140 |
| **PEA** | **0.96** | **0.45** | **0.06 – 1.85** | **50** | **2.14** | **0.038** |
| Group*PEA | -0.24 | 0.15 | -0.54 – 0.06 | 50 | -1.64 | 0.108 |
| Sex | 0.01 | 1.07 | -2.14 – 2.16 | 50 | 0.01 | 0.990 |
| **Age** | **0.12** | **0.05** | **0.03 – 0.22** | **50** | **2.68** | **0.010** |
| Cannabis use | 0.07 | 0.19 | -0.30 – 0.45 | 50 | 0.40 | 0.693 |
| PEA*Sex | -0.04 | 0.17 | -0.37 – 0.29 | 50 | -0.24 | 0.815 |
| PEA*Age | -0.02 | 0.01 | -0.04 – 0.003 | 50 | -1.75 | 0.087 |
| Group*Sex | 0.09 | 0.42 | -0.76 – 0.93 | 50 | 0.20 | 0.841 |
| Group*Age | -0.01 | 0.02 | -0.06 – 0.03 | 50 | -0.59 | 0.555 |

Dependent variable: *change score negative affect (NA)*

|  | Estimate | SE | CI | df | t | *p* |
| --- | --- | --- | --- | --- | --- | --- |
| (Intercept) | 2.72 | 1.21 | 0.30 – 5.14 | 50 | 2.26 | 0.028 |
| **Group** | **-1.21** | **0.56** | **-2.34 – -0.08** | **50** | **-2.15** | **0.037** |
| PEA | -0.33 | 0.20 | -0.73 – 0.07 | 50 | -1.65 | 0.105 |
| Group*PEA | 0.10 | 0.07 | -0.04 – 0.23 | 50 | 1.46 | 0.152 |
| Sex | -0.07 | 0.48 | -1.02 – 0.89 | 50 | -0.14 | 0.892 |
| **Age** | **-0.06** | **0.02** | **-0.10 – -0.02** | **50** | **-2.81** | **0.007** |
| Cannabis use | 0.06 | 0.08 | -0.11 – 0.22 | 50 | 0.71 | 0.484 |
| PEA*Sex | -0.03 | 0.07 | -0.18 – 0.12 | 50 | -0.38 | 0.704 |
| PEA*Age | 0.01 | 0.01 | -0.002 – 0.02 | 50 | 1.49 | 0.143 |
| Group*Sex | 0.20 | 0.19 | -0.18 – 0.57 | 50 | 1.05 | 0.300 |
| Group*Age | 0.01 | 0.01 | -0.01 – 0.03 | 50 | 1.17 | 0.246 |

Abbreviations: anandamide (AEA), oleoylethanolamide (OEA), palmitoylethanolamide (PEA)

**Table S7a** Linear mixed model parameters with the fixed factor AEA, GROUP, and AEA*GROUP interactions controlled for AGE, SEX, their interactions, and depression (BDI).

Dependent variable: *estimated* *percentage ball received*

|  | Estimate | SE | CI | df | t | *p* |
| --- | --- | --- | --- | --- | --- | --- |
| (Intercept) | 33.87 | 32.43 | -31.27 – 99.02 | 50 | 1.04 | 0.301 |
| Group | -16.38 | 17.42 | -51.37 – 18.61 | 50 | -0.94 | 0.352 |
| AEA | -41.48 | 36.81 | -115.41 – 32.45 | 50 | -1.13 | 0.265 |
| **Group*AEA** | **42.05** | **13.33** | **15.28 – 68.81** | **50** | **3.16** | **0.003** |
| Sex | 0.51 | 12.49 | -24.58 – 25.60 | 50 | 0.04 | 0.968 |
| Age | 0.84 | 0.44 | -0.05 – 1.73 | 50 | 1.89 | 0.064 |
| BDI | -0.02 | 0.20 | -0.42 – 0.38 | 50 | -0.10 | 0.918 |
| AEA*Sex | 11.41 | 13.17 | -15.05 – 37.86 | 50 | 0.87 | 0.391 |
| AEA*Age | -1.00 | 0.63 | -2.26 – 0.26 | 50 | -1.59 | 0.118 |
| Group*Sex | -9.26 | 5.47 | -20.24 – 1.73 | 50 | -1.69 | 0.097 |
| Group*Age | -0.14 | 0.33 | -0.80 – 0.51 | 50 | -0.44 | 0.662 |

Dependent variable: *feeling included*

|  | Estimate | SE | CI | df | t | *p* |
| --- | --- | --- | --- | --- | --- | --- |
| (Intercept) | 9.32 | 5.35 | -1.42 – 20.07 | 50 | 1.74 | 0.088 |
| Group | -0.89 | 2.87 | -6.66 – 4.89 | 50 | -0.31 | 0.759 |
| AEA | -11.84 | 6.07 | -24.03 – 0.36 | 50 | -1.95 | 0.057 |
| **Group*AEA** | **5.85** | **2.20** | **1.43 – 10.26** | **50** | **2.66** | **0.010** |
| Sex | -2.11 | 2.06 | -6.25 – 2.03 | 50 | -1.02 | 0.311 |
| Age | 0.13 | 0.07 | -0.02 – 0.27 | 50 | 1.74 | 0.087 |
| BDI | -0.02 | 0.03 | -0.08 – 0.05 | 50 | -0.54 | 0.593 |
| **AEA*Sex** | **4.49** | **2.17** | **0.13 – 8.85** | **50** | **2.07** | **0.044** |
| AEA*Age | -0.06 | 0.10 | -0.27 – 0.15 | 50 | -0.57 | 0.574 |
| Group*Sex | -1.77 | 0.90 | -3.58 – 0.05 | 50 | -1.96 | 0.056 |
| Group*Age | -0.07 | 0.05 | -0.18 – 0.04 | 50 | -1.25 | 0.218 |

Dependent variable: *feeling excluded*

|  | Estimate | SE | CI | df | t | *p* |
| --- | --- | --- | --- | --- | --- | --- |
| (Intercept) | 0.97 | 7.10 | -13.29 – 15.24 | 50 | 0.14 | 0.892 |
| Group | 0.95 | 3.82 | -6.71 – 8.62 | 50 | 0.25 | 0.804 |
| AEA | 12.33 | 8.06 | -3.87 – 28.52 | 50 | 1.53 | 0.133 |
| Group*AEA | -4.97 | 2.92 | -10.83 – 0.90 | 50 | -1.70 | 0.095 |
| Sex | 2.61 | 2.74 | -2.89 – 8.10 | 50 | 0.95 | 0.345 |
| Age | -0.10 | 0.10 | -0.29 – 0.10 | 50 | -0.98 | 0.331 |
| BDI | 0.00 | 0.04 | -0.09 – 0.09 | 50 | -0.01 | 0.994 |
| AEA*Sex | -4.78 | 2.89 | -10.57 – 1.02 | 50 | -1.66 | 0.104 |
| AEA*Age | -0.02 | 0.14 | -0.30 – 0.26 | 50 | -0.14 | 0.892 |
| Group*Sex | 1.10 | 1.20 | -1.31 – 3.50 | 50 | 0.91 | 0.365 |
| Group*Age | 0.07 | 0.07 | -0.07 – 0.21 | 50 | 0.97 | 0.338 |

Dependent variable: *change score positive affect (PA)*

|  | Estimate | SE | CI | df | t | *p* |
| --- | --- | --- | --- | --- | --- | --- |
| (Intercept) | -4.24 | 2.58 | -9.43 – 0.94 | 50 | -1.64 | 0.107 |
| Group | 2.46 | 1.39 | -0.33 – 5.24 | 50 | 1.77 | 0.082 |
| AEA | 2.84 | 2.93 | -3.04 – 8.73 | 50 | 0.97 | 0.336 |
| Group*AEA | -1.36 | 1.06 | -3.49 – 0.77 | 50 | -1.28 | 0.205 |
| Sex | -0.14 | 0.99 | -2.13 – 1.86 | 50 | -0.14 | 0.891 |
| **Age** | **0.08** | **0.04** | **0.01 – 0.15** | **50** | **2.14** | **0.037** |
| BDI | -0.01 | 0.02 | -0.04 – 0.03 | 50 | -0.34 | 0.736 |
| AEA*Sex | -0.30 | 1.05 | -2.41 – 1.80 | 50 | -0.29 | 0.773 |
| AEA*Age | -0.01 | 0.05 | -0.11 – 0.10 | 50 | -0.12 | 0.909 |
| Group*Sex | 0.11 | 0.44 | -0.77 – 0.98 | 50 | 0.25 | 0.802 |
| Group*Age | -0.04 | 0.03 | -0.09 – 0.01 | 50 | -1.62 | 0.112 |

Dependent variable: *change score negative affect (NA)*

|  | Estimate | SE | CI | df | t | *p* |
| --- | --- | --- | --- | --- | --- | --- |
| (Intercept) | 1.85 | 1.14 | -0.43 – 4.14 | 50 | 1.63 | 0.110 |
| Group | -1.19 | 0.61 | -2.42 – 0.04 | 50 | -1.94 | 0.058 |
| AEA | -0.43 | 1.29 | -3.03 – 2.17 | 50 | -0.33 | 0.741 |
| Group*AEA | 0.07 | 0.47 | -0.87 – 1.01 | 50 | 0.16 | 0.875 |
| Sex | 0.00 | 0.44 | -0.88 – 0.89 | 50 | 0.01 | 0.993 |
| **Age** | -0.05 | 0.02 | **-0.08 – -0.02** | **50** | -2.96 | 0.005 |
| BDI | 0.00 | 0.01 | -0.01 – 0.02 | 50 | 0.63 | 0.529 |
| AEA*Sex | -0.24 | 0.46 | -1.16 – 0.69 | 50 | -0.51 | 0.614 |
| AEA*Age | 0.01 | 0.02 | -0.03 – 0.05 | 50 | 0.45 | 0.653 |
| Group*Sex | 0.24 | 0.19 | -0.15 – 0.62 | 50 | 1.23 | 0.226 |
| **Group*Age** | 0.02 | 0.01 | **0.001 – 0.05** | **50** | 2.11 | 0.040 |

**Table S7b** Linear mixed model parameters with the fixed factor OEA, GROUP, and OEA*GROUP interactions controlled for AGE, SEX, their interactions, and depression (BDI).

Dependent variable: *estimated* *percentage ball received*

|  | Estimate | SE | CI | df | t | *p* |
| --- | --- | --- | --- | --- | --- | --- |
| (Intercept) | 27.08 | 29.34 | -31.86 – 86.01 | 50 | 0.92 | 0.361 |
| Group | -13.64 | 16.74 | -47.27 – 19.99 | 50 | -0.82 | 0.419 |
| OEA | -13.22 | 14.23 | -41.81 – 15.37 | 50 | -0.93 | 0.358 |
| **Group*OEA** | **16.30** | **4.69** | **6.88 – 25.72** | **50** | **3.47** | **0.001** |
| Sex | 2.63 | 11.27 | -20.01 – 25.26 | 50 | 0.23 | 0.817 |
| **Age** | **0.81** | **0.40** | **0.002 – 1.61** | **50** | **2.01** | **0.049** |
| BDI | -0.09 | 0.20 | -0.50 – 0.32 | 50 | -0.45 | 0.657 |
| OEA*Sex | 1.89 | 5.20 | -8.56 – 12.33 | 50 | 0.36 | 0.718 |
| OEA*Age | -0.40 | 0.24 | -0.87 – 0.08 | 50 | -1.69 | 0.098 |
| Group*Sex | -6.80 | 5.34 | -17.52 – 3.92 | 50 | -1.27 | 0.208 |
| Group*Age | -0.17 | 0.32 | -0.81 – 0.47 | 50 | -0.53 | 0.599 |

Dependent variable: *feeling included*

|  | Estimate | SE | CI | df | t | *p* |
| --- | --- | --- | --- | --- | --- | --- |
| (Intercept) | 9.20 | 5.03 | -0.91 – 19.31 | 50 | 1.83 | 0.073 |
| Group | 1.57 | 2.87 | -4.20 – 7.33 | 50 | 0.55 | 0.588 |
| **OEA** | **-5.75** | **2.44** | **-10.65 – -0.85** | **50** | **-2.36** | **0.022** |
| **Group*OEA** | **1.72** | **0.80** | **0.10 – 3.34** | **50** | **2.14** | **0.037** |
| Sex | -2.41 | 1.93 | -6.29 – 1.48 | 50 | -1.24 | 0.219 |
| Age | 0.10 | 0.07 | -0.04 – 0.24 | 50 | 1.48 | 0.147 |
| BDI | -0.04 | 0.04 | -0.11 – 0.03 | 50 | -1.22 | 0.230 |
| **OEA*Sex** | **2.02** | **0.89** | **0.23 – 3.81** | **50** | **2.26** | **0.028** |
| OEA*Age | 0.02 | 0.04 | -0.06 – 0.10 | 50 | 0.42 | 0.679 |
| Group*Sex | -1.58 | 0.92 | -3.42 – 0.26 | 50 | -1.73 | 0.091 |
| Group*Age | -0.10 | 0.06 | -0.21 – 0.01 | 50 | -1.86 | 0.069 |

Dependent variable: *feeling excluded*

|  | Estimate | SE | CI | df | t | *p* |
| --- | --- | --- | --- | --- | --- | --- |
| (Intercept) | 4.33 | 6.84 | -9.41 – 18.07 | 50 | 0.63 | 0.530 |
| Group | -0.71 | 3.90 | -8.54 – 7.13 | 50 | -0.18 | 0.857 |
| OEA | 3.61 | 3.32 | -3.05 – 10.27 | 50 | 1.09 | 0.282 |
| Group*OEA | -1.39 | 1.09 | -3.58 – 0.81 | 50 | -1.27 | 0.210 |
| Sex | 1.18 | 2.63 | -4.10 – 6.46 | 50 | 0.45 | 0.655 |
| Age | -0.08 | 0.09 | -0.27 – 0.11 | 50 | -0.88 | 0.382 |
| BDI | 0.02 | 0.05 | -0.08 – 0.11 | 50 | 0.38 | 0.709 |
| OEA*Sex | -1.35 | 1.21 | -3.78 – 1.08 | 50 | -1.11 | 0.271 |
| OEA*Age | -0.01 | 0.06 | -0.12 – 0.10 | 50 | -0.17 | 0.862 |
| Group*Sex | 1.05 | 1.24 | -1.45 – 3.55 | 50 | 0.84 | 0.404 |
| Group*Age | 0.07 | 0.07 | -0.08 – 0.22 | 50 | 0.89 | 0.380 |

Dependent variable: *change score positive affect (PA)*

|  | Estimate | SE | CI | df | t | *p* |
| --- | --- | --- | --- | --- | --- | --- |
| (Intercept) | -4.03 | 2.41 | -8.87 – 0.81 | 50 | -1.67 | 0.101 |
| Group | 2.53 | 1.38 | -0.23 – 5.29 | 50 | 1.84 | 0.072 |
| OEA | 0.99 | 1.17 | -1.36 – 3.33 | 50 | 0.84 | 0.403 |
| Group*OEA | -0.56 | 0.39 | -1.33 – 0.22 | 50 | -1.45 | 0.153 |
| Sex | 0.00 | 0.93 | -1.86 – 1.86 | 50 | 0.00 | 0.998 |
| Age | 0.07 | 0.03 | 0.001 – 0.13 | 50 | 2.03 | 0.048 |
| BDI | 0.00 | 0.02 | -0.04 – 0.03 | 50 | -0.21 | 0.835 |
| OEA*Sex | -0.11 | 0.43 | -0.97 – 0.75 | 50 | -0.26 | 0.798 |
| OEA*Age | 0.00 | 0.02 | -0.03 – 0.04 | 50 | 0.23 | 0.817 |
| Group*Sex | 0.03 | 0.44 | -0.85 – 0.91 | 50 | 0.06 | 0.953 |
| Group*Age | -0.05 | 0.03 | -0.10 – 0.01 | 50 | -1.74 | 0.088 |

Dependent variable: *change score negative affect (NA)*

|  | Estimate | SE | CI | df | t | *p* |
| --- | --- | --- | --- | --- | --- | --- |
| (Intercept) | 1.60 | 1.09 | -0.59 – 3.79 | 50 | 1.47 | 0.148 |
| Group | -1.20 | 0.62 | -2.45 – 0.05 | 50 | -1.93 | 0.060 |
| OEA | -0.06 | 0.53 | -1.12 – 1.00 | 50 | -0.11 | 0.911 |
| Group*OEA | 0.04 | 0.17 | -0.31 – 0.39 | 50 | 0.24 | 0.811 |
| Sex | -0.04 | 0.42 | -0.88 – 0.80 | 50 | -0.10 | 0.924 |
| **Age** | **-0.04** | **0.02** | **-0.07 – -0.01** | **50** | **-2.64** | **0.011** |
| BDI | 0.01 | 0.01 | -0.01 – 0.02 | 50 | 0.78 | 0.439 |
| OEA*Sex | -0.11 | 0.19 | -0.49 – 0.28 | 50 | -0.55 | 0.585 |
| OEA*Age | 0.00 | 0.01 | -0.02 – 0.02 | 50 | 0.25 | 0.806 |
| Group*Sex | 0.25 | 0.20 | -0.15 – 0.65 | 50 | 1.25 | 0.218 |
| Group*Age | 0.02 | 0.01 | 0.001 – 0.05 | 50 | 1.95 | 0.057 |

**Table S7c** Linear mixed model parameters with the fixed factor PEA, GROUP, and PEA*GROUP interactions controlled for AGE, SEX, their interactions, and depression (BDI).

Dependent variable: *estimated* *percentage ball received*

|  | Estimate | SE | CI | df | t | *p* |
| --- | --- | --- | --- | --- | --- | --- |
| (Intercept) | -10.17 | 38.53 | -87.55 – 67.21 | 50 | -0.26 | 0.793 |
| Group | 9.42 | 18.20 | -27.14 – 45.97 | 50 | 0.52 | 0.607 |
| PEA | -0.32 | 6.53 | -13.44 – 12.80 | 50 | -0.05 | 0.961 |
| Group*PEA | 2.50 | 2.07 | -1.65 – 6.66 | 50 | 1.21 | 0.232 |
| Sex | 13.63 | 15.59 | -17.69 – 44.95 | 50 | 0.87 | 0.386 |
| Age | 0.66 | 0.65 | -0.64 – 1.97 | 50 | 1.03 | 0.310 |
| BDI | 0.02 | 0.23 | -0.44 – 0.48 | 50 | 0.10 | 0.923 |
| PEA*Sex | -1.35 | 2.52 | -6.40 – 3.71 | 50 | -0.54 | 0.595 |
| PEA*Age | 0.00 | 0.14 | -0.29 – 0.29 | 50 | -0.01 | 0.994 |
| Group*Sex | -5.97 | 6.02 | -18.05 –6.11 | 50 | -0.99 | 0.326 |
| Group*Age | -0.50 | 0.33 | -1.16 – 0.17 | 50 | -1.50 | 0.140 |

Dependent variable: *feeling included*

|  | Estimate | SE | CI | df | t | *p* |
| --- | --- | --- | --- | --- | --- | --- |
| (Intercept) | -0.64 | 6.41 | -13.50 – 12.23 | 50 | -0.10 | 0.921 |
| Group | 3.98 | 3.03 | -2.10 – 10.06 | 50 | 1.32 | 0.194 |
| PEA | -0.16 | 1.09 | -2.34 – 2.02 | 50 | -0.15 | 0.884 |
| Group*PEA | 0.08 | 0.34 | -0.61 – 0.77 | 50 | 0.23 | 0.818 |
| Sex | 1.37 | 2.59 | -3.84 – 6.58 | 50 | 0.53 | 0.599 |
| Age | 0.05 | 0.11 | -0.16 – 0.27 | 50 | 0.51 | 0.615 |
| BDI | -0.01 | 0.04 | -0.09 – 0.07 | 50 | -0.27 | 0.786 |
| PEA*Sex | -0.04 | 0.42 | -0.88 – 0.80 | 50 | -0.09 | 0.926 |
| PEA*Age | 0.02 | 0.02 | -0.03 – 0.06 | 50 | 0.63 | 0.529 |
| Group*Sex | -1.32 | 1.00 | -3.33 – 0.69 | 50 | -1.32 | 0.194 |
| **Group*Age** | -0.10 | 0.06 | -0.21 – 0.01 | 50 | -1.86 | 0.068 |

Dependent variable: *feeling excluded*

|  | Estimate | SE | CI | df | t | *p* |
| --- | --- | --- | --- | --- | --- | --- |
| (Intercept) | 6.73 | 8.47 | -10.29 – 23.75 | 50 | 0.80 | 0.431 |
| Group | -1.32 | 4.00 | -9.35 – 6.72 | 50 | -0.33 | 0.744 |
| PEA | 0.56 | 1.44 | -2.32 – 3.45 | 50 | 0.39 | 0.697 |
| Group*PEA | -0.16 | 0.46 | -1.08 – 0.75 | 50 | -0.35 | 0.724 |
| Sex | 0.42 | 3.43 | -6.46 – 7.31 | 50 | 0.12 | 0.902 |
| Age | -0.04 | 0.14 | -0.33 – 0.25 | 50 | -0.28 | 0.781 |
| BDI | 0.00 | 0.05 | -0.10 – 0.10 | 50 | 0.03 | 0.980 |
| PEA*Sex | -0.22 | 0.55 | -1.33 – 0.89 | 50 | -0.40 | 0.691 |
| PEA*Age | -0.01 | 0.03 | -0.07 – 0.06 | 50 | -0.23 | 0.823 |
| Group*Sex | 0.61 | 1.32 | -2.05 – 3.27 | 50 | 0.46 | 0.646 |
| Group*Age | 0.05 | 0.07 | -0.10 – 0.20 | 50 | 0.68 | 0.503 |

Dependent variable: *change score positive affect (PA)*

|  | Estimate | SE | CI | df | t | *p* |
| --- | --- | --- | --- | --- | --- | --- |
| (Intercept) | -6.07 | 2.74 | -11.58 – -0.57 | 50 | -2.22 | 0.031 |
| Group | 1.96 | 1.29 | -0.64 – 4.56 | 50 | 1.52 | 0.135 |
| PEA | 0.92 | 0.47 | -0.02 – 1.85 | 50 | 1.98 | 0.054 |
| Group*PEA | -0.24 | 0.15 | -0.53 – 0.06 | 50 | -1.60 | 0.115 |
| Sex | -0.02 | 1.11 | -2.25 – 2.21 | 50 | -0.02 | 0.986 |
| **Age** | **0.12** | **0.05** | **0.03 – 0.22** | **50** | **2.68** | **0.010** |
| BDI | -0.01 | 0.02 | -0.04 – 0.03 | 50 | -0.28 | 0.779 |
| PEA*Sex | -0.03 | 0.18 | -0.39 – 0.33 | 50 | -0.16 | 0.872 |
| PEA*Age | -0.02 | 0.01 | -0.04 – 0.003 | 50 | -1.71 | 0.094 |
| Group*Sex | 0.05 | 0.43 | -0.81 – 0.91 | 50 | 0.12 | 0.907 |
| Group*Age | -0.02 | 0.02 | -0.06 – 0.03 | 50 | -0.65 | 0.518 |

Dependent variable: *change score negative affect (NA)*

|  | Estimate | SE | CI | df | t | *p* |
| --- | --- | --- | --- | --- | --- | --- |
| (Intercept) | 2.61 | 1.21 | 0.18 – 5.05 | 50 | 2.16 | 0.036 |
| **Group** | **-1.32** | **0.57** | **-2.47 – -0.17** | **50** | **-2.31** | **0.025** |
| PEA | -0.28 | 0.21 | -0.69 – 0.13 | 50 | -1.36 | 0.180 |
| Group*PEA | 0.10 | 0.07 | -0.03 – 0.23 | 50 | 1.48 | 0.144 |
| Sex | 0.11 | 0.49 | -0.88 – 1.10 | 50 | 0.22 | 0.823 |
| **Age** | **-0.06** | **0.02** | **-0.10 – -0.02** | **50** | **-2.91** | **0.005** |
| BDI | 0.01 | 0.01 | -0.01 – 0.02 | 50 | 0.92 | 0.364 |
| PEA*Sex | -0.07 | 0.08 | -0.22 – 0.09 | 50 | -0.82 | 0.415 |
| PEA*Age | 0.01 | 0.01 | -0.002 – 0.02 | 50 | 1.60 | 0.115 |
| Group*Sex | 0.22 | 0.19 | -0.16 – 0.60 | 50 | 1.19 | 0.242 |
| Group*Age | 0.01 | 0.01 | -0.01 – 0.03 | 50 | 1.20 | 0.237 |

Abbreviations: anandamide (AEA), Beck Depression Inventory (BDI), oleoylethanolamide (OEA), palmitoylethanolamide (PEA)


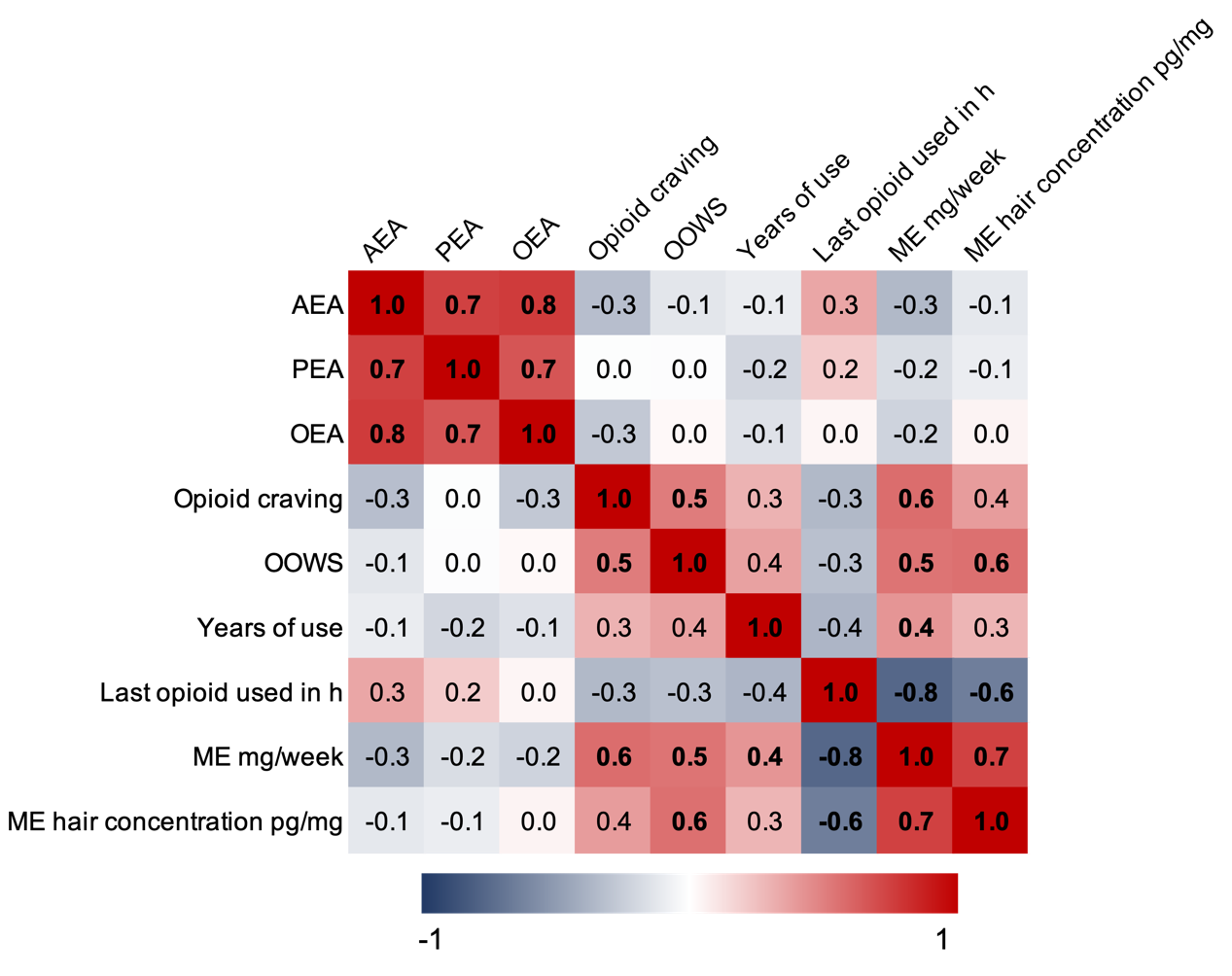


**Fig. S1 Spearman correlations between *N*-acylethanolamines and opioid use variables.**

Heat matrix of correlation coefficients with significant correlations shown in bold (*p*<.05) within the NMPOU group.

Abbreviations: anandamide (AEA), Morphine equivalence (ME), oleoylethanolamide (OEA), Objective opioid withdrawal symptoms (OOWS), palmitoylethanolamide (PEA)


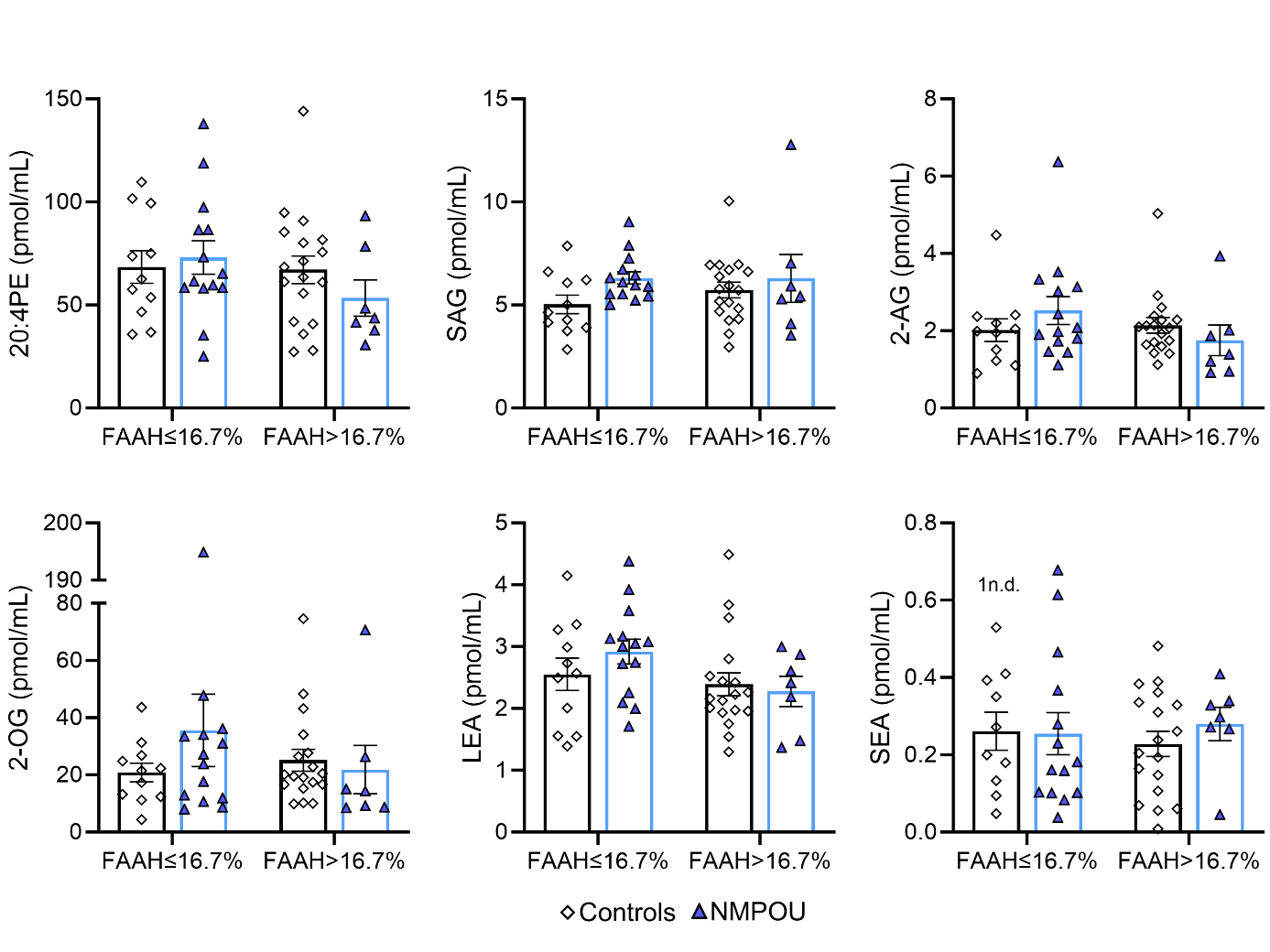


**Fig. S2 Quantified plasma lipids sorted by FAAH activity.**

Quantified plasma lipid levels sorted by FAAH activity measured in whole blood (see Fig. 3b, FAAH specific ^3^H-AEA hydrolysis). Data are presented as scatter dot plot with bar showing mean ± SEM. Each dot represents the quantified value of one participant. For statistical analysis a two-tailed, unpaired t-test was performed with a *p*-value <0.05 considered to indicate a significant difference between the two compared groups. Abbrevations: 20:4PE, 1,2-diarachidonoyl-sn-glycero-3-phosphoethanolamine; 2-AG, 2-arachidonoylglycerol; 2-OG, 2-oleoylglycerol; LEA, linoleoyl ethanolamide; SAG, 1-Stearoyl-2-arachidonoyl-sn-glycerol; SEA, stearoyl ethanolamide.

**References**

1 Reynoso-Moreno I, Tietz S, Vallini E, Engelhardt B, Gertsch J, Chicca A. Selective Endocannabinoid Reuptake Inhibitor WOBE437 Reduces Disease Progression in a Mouse Model of Multiple Sclerosis. ACS Pharmacol Transl Sci. 2021;4(2):765-79.

2 Hillard CJ. Circulating Endocannabinoids: From Whence Do They Come and Where are They Going? Neuropsychopharmacology. 2018;43(1):155-72.
